# Quantitative Evaluation and Optimization of Museum Fatigue Using Computer Vision Human Pose Estimation

**DOI:** 10.3390/s26020729

**Published:** 2026-01-21

**Authors:** Zhongsu Cheng, Yuxiao Zhang, Lin Zhang

**Affiliations:** 1 Institute of Design and Research, Yangtze University, Jingzhou 434100, China; zhongsuc@gmail.com; 2 School of Environmental Art, Hubei Institute of Fine Arts, Wuhan 430205, China; 3 School of Computer Sciences, Yangtze University, Jingzhou 434100, China; 18966892476@163.com; 4 School of Urban Construction, Yangtze University, Jingzhou 434100, China

**Keywords:** museum, computer vision, fatigue mitigation, cognitive load

## Abstract

Museums are key institutions for cultural communication and public education, and their operating concept is shifting from exhibit-centered to experience-centered. As expectations for exhibition experience rise, museum fatigue has become a major constraint on visitors. Existing studies rely on questionnaires and other subjective measures, which makes it difficult to locate fatigue in specific spaces. At the same time, body pose detection and fatigue recognition techniques remain hard to apply in museums because of complex spatial configurations and dense visitor flows. Effective methods for quantifying and mitigating museum fatigue are still lacking. This study proposes a contact-free sensing scheme based on computer vision and builds a coupled analytical framework with three stages: Human Pose Estimation (HPE) for visitor posture detection, fatigue assessment, and fatigue mitigation. A Fatigue Index (FI) quantifies bodily fatigue. Applying this index to the exhibition space in both the baseline and adjusted configurations guides the formulation of mitigation strategies and shows a consistent reduction in FI, which indicates that the adopted measures are effective. The proposed approach establishes a complete frame from fatigue quantification to fatigue mitigation, supports evaluation of exhibition space design, and provides theoretical and methodological support for future improvements to museum experience.

## 1. Introduction

Museums act as major venues for cultural communication and public education. In the context of the so-called “experience economy” and expanding cultural consumption, they are moving away from static, exhibit-centered display toward spatial settings that give more weight to visitor agency and to the overall quality of the visit [1]. During this transition, visitors tend to stay longer in the galleries, confront denser streams of information, and interact with a broader range of media. Within such conditions, “museum fatigue” has attracted increasing attention as a combined physical and mental load associated with visiting. It is typically expressed as declining attention, reduced engagement, and lower overall satisfaction, which in turn weakens both the continuity of the visit and its cognitive outcomes [2]. To achieve a substantive improvement in exhibition experience, two interrelated questions must be addressed. The first is how to quantify museum fatigue under real visiting conditions in a scientifically sound way that can be aligned with specific spatial locations. The second is how to translate these quantitative results into feasible and assessable mitigation strategies that can feed back into the design of exhibition spaces.

In conceptual terms, museum fatigue can be understood as a consequence of how visitors process information while moving through the galleries. Cognitive Load Theory describes working memory as a narrow bottleneck: only a limited amount of material can be handled at one time. When the incoming stream of information goes beyond this bottleneck, task performance tends to fall, and the activity is experienced as more tiring and mentally demanding. Although the theory originated in educational psychology, it has proved highly informative in settings that require sustained attention and intensive information processing. Following Sweller, this study adopts a three-part distinction of cognitive load. Intrinsic load refers to difficulty built into the task itself, extraneous load arises from the way information is arranged and presented, and germane load denotes the share of mental effort that actually supports the formation and refinement of schemas [3]. Paas and colleagues proposed practical procedures for measuring cognitive load by combining ratings of perceived effort with performance indicators, and used these procedures to compare different instructional and interface conditions [4]. Mayer later placed this framework in the context of multimedia and learning environment design. Through controlled studies, he showed that choices about the sequencing and modality of information, and about how text and images are combined, can be used to manage cognitive load in a planned way [5]. Following this line of reasoning, the present study treats museum fatigue as emerging from the interaction between cognitive load and the spatial, as well as narrative, conditions of the exhibition. Therefore, any quantitative account of fatigue needs to keep this link to the visitor experience explicit.

In empirical work, questionnaires remain one of the most frequently used tools in museum audience studies and in the evaluation of fatigue. They are mainly used to describe psychological dimensions such as attention, interest, satisfaction, and self-perceived fatigue [6,7]. Common instruments include the Semantic Differential (SD) scale and the Likert scale. More recent studies also make use of overall experience scores and user experience scales to examine how exhibition format, degree of interaction, and type of guiding medium shape visitor perceptions [8,9]. Many contributions incorporate demographic variables such as sex, age, frequency of visits, and visiting motives, and conduct stratified analyses to reveal group differences in spatial use and fatigue-related experience [6,10]. Earlier work relied largely on exit surveys to obtain an overall judgment at the end of the visit [8]. Later studies introduced short in situ micro-surveys placed beside exhibits and combined them with behavioral tracking, so as to capture momentary perception in specific locations and compare it with flow paths and dwell time [11].

However, questionnaires on their own have several built-in limitations when the aim is to uncover the spatiotemporal pattern of museum fatigue. First, as essentially retrospective tools, they are not well-suited to describing the dynamic and continuous evolution of fatigue during a visit. Second, the findings depend strongly on self-report and recall, and are therefore vulnerable to social desirability bias, memory blur, and transient mood, which restricts the rigor of the results. Third, responses are usually aggregated at the level of an exhibition or gallery, which makes it difficult to align the data with specific spatial nodes and environmental features and to derive finely targeted spatial interventions [2]. These constraints have prompted researchers to turn to fatigue-detection methods that are already well established in other domains and to explore how such techniques might be adapted to museum settings.

In industrial settings, body posture analysis usually focuses on variables such as joint angles, postural stability, repetition count, and task duration. These measures are often combined with ergonomic risk scores such as RULA and REBA [12], and with indices of local muscle loading to build models of cumulative musculoskeletal fatigue. Recent studies add composite features that span time and frequency domains [13], indices of coactivation in antagonist muscle pairs, and the amplitude of small postural sway. They also employ sequence models such as hidden Markov and hidden semi-Markov models, temporal convolutional networks, and graph-based networks on skeletal data, including ST GCN and 2 s AGCN, to describe fatigue patterns across multiple joints [14]. Surface electromyography (EMG) and measures of postural stability, for example, center of pressure (COP), are used as external references for validation [15,16]. However, these approaches are designed for standardized work cycles with repetitive tasks at fixed workstations, and their outputs are expressed as discrete risk levels. When they are transferred directly to open museum environments with freely moving visitors and self-directed behavior, their underlying assumptions no longer fit well.

In clinical rehabilitation and geriatric assessment, research is centered on quantitative evaluation of gait and balance. Keypoint tracking is used to extract temporal and spatial gait parameters such as cadence, step length, swing and stance phases, and symmetry, along with indicators of static and dynamic postural stability, including the distance between the body center of mass and the footprint projection or the Romberg ratio [17]. In many studies, these quantitative descriptors are interpreted together with established clinical functional scales, including the Berg Balance Scale (BBS), Timed Up and Go (TUG), and the Functional Reach (FRT) test, so that gait and balance parameters can be related to established clinical judgments [18]. Methodological studies that combine steady state and transitional phases and that identify small loss of balance events have improved sensitivity to early functional decline and instability [19]. However, the primary objective in this field is to detect pathological impairment and risk of falls. Experiments are usually conducted on dedicated walkways or in laboratory environments that are set up to highlight atypical gait patterns [20]. By contrast, fatigue in museum visitors is a normal physical and psychological response that develops in the course of cultural experience [2]. Its causes, thresholds of concern, and evaluation targets differ fundamentally from those in clinical settings. If clinical indices and thresholds are applied without such distinctions, fatigue is likely to be overstated or even misinterpreted.

In sports science and training monitoring, posture and motion analysis are used to describe how fatigue develops, together with technical degradation over time. Studies extract time and frequency domain features and input them into sequence models such as temporal convolutional networks, bidirectional long short-term memory networks, and Transformer-based architectures, in order to quantify subtle changes in movement as fatigue accumulates [21]. External variables, including power output, technical scores, and measures of movement consistency, serve as reference points for interpretation [22]. More recent work introduces attention mechanisms and multi-scale sliding windows to pinpoint the time segments in which the technique begins to deteriorate [23]. At first sight, this strand of research may seem relevant because it also traces how movement form changes over time. However, in competitive or training contexts, fatigue usually develops under very heavy or near-maximal load, and it is often accompanied by abrupt drops in performance indicators [24]. In museum visits, fatigue accumulates more slowly during extended periods of gentle walking and standing, and the outward changes in posture are far less pronounced. Changes in posture are modest, and the “task” does not follow a fixed and well-defined movement pattern. Therefore, models and thresholds developed in sports contexts do not transfer well when the aim is to identify low-intensity cumulative fatigue in museum spaces [2].

Taken together, approaches to posture and fatigue in industrial, clinical, and sports contexts are deeply rooted in their own task structures and environmental assumptions. These assumptions sit uneasily with the open, low-intensity, and highly self-directed character of museum visits. As a result, current research on museum fatigue still faces a methodological gap. There is no quantitative diagnostic tool that can, at the same time, accommodate unstructured visiting behavior, capture subtle compensatory movements at low intensity, and remain dynamically linked to the exhibition space. This gap, combined with the limitations of questionnaire-based methods, forms the basis for the computer vision-based, contact-free framework for museum fatigue quantification proposed in this study.

**Figure 1:**
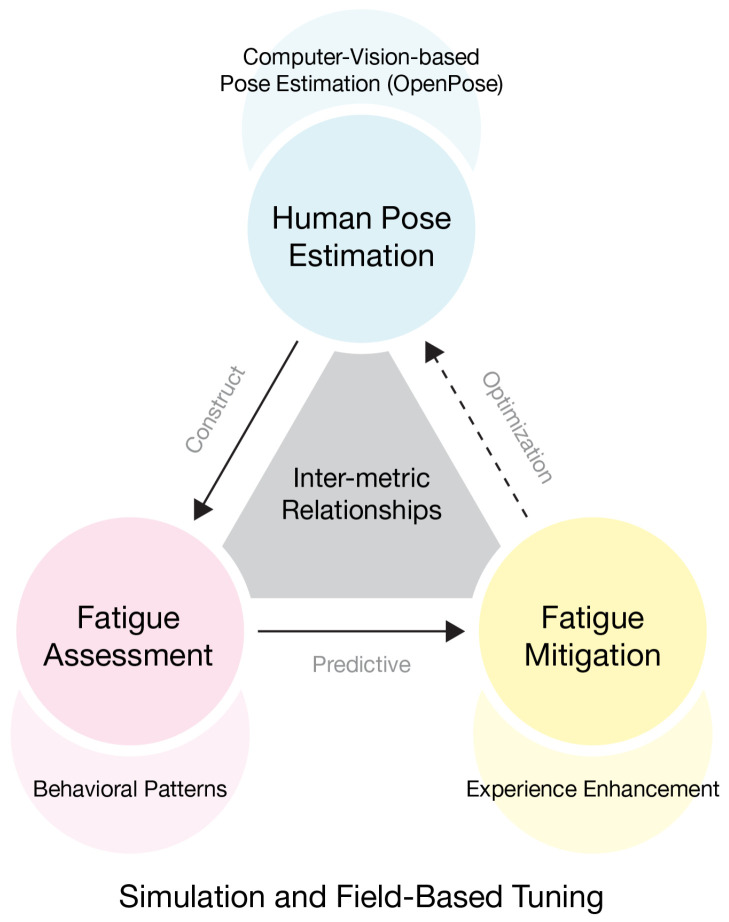
Conceptual framework of computer-vision-based fatigue assessment and mitigation.

Building on these issues, the present work proceeds from two related questions: how to quantify fatigue, and how to use this quantitative description to derive mitigation strategies. First, it introduces Human Pose Estimation (HPE) based on computer vision to establish a contact-free, continuous, and spatially precise pathway for fatigue quantification that is tailored to museum settings. Second, it develops an analytical structure that connects pose estimation, fatigue assessment, and fatigue mitigation, as shown in Figure 1.

The methodological framework is motivated by a combined consideration of real-world constraints in museums and the goal of a scalable application. For long-term, unobtrusive monitoring of visitor populations, conventional physiological sensing solutions (e.g., surface electromyography and heart-rate monitoring) can provide more direct physiological references; however, in real viewing contexts, they are often constrained by wearing compliance, management overhead, and the potential to interfere with natural behaviors, making routine, large-scale deployment difficult. Accordingly, this study focuses on population-level observable behaviors as proxy signals and establishes an associative representation between spatial characteristics and overall fatigue risk. Under this positioning, the central contribution of this work is to propose and validate an alternative quantification framework that is fully grounded in observable behaviors, biomechanically interpretable, and suitable for real museum visits.

Given that a unified benchmark for continuous, ecological measurement of “museum fatigue” remains to be established [25], this work therefore concentrates on systematically examining the effectiveness of the proposed framework and its mechanistic feasibility in complex real-world environments. To this end, we adopt a triangulation research design [26], integrating computer-vision-derived behavioral features, questionnaire feedback, and pre-/post-optimization evaluation results to construct a mutually corroborating evidence chain across construct validity, predictive validity, and optimization validity, thereby assessing the explanatory power and effectiveness of the framework in situ.

## 2. Methodological Framework

### 2.1. Research Focus and System Architecture

“Museum fatigue” is not a single phenomenon but a composite condition arising from the interaction of multiple processes, including physical fatigue, cognitive load, and decision fatigue [25]. This study focuses on the physiological fatigue induced by prolonged physical or mental exertion during museum visits and its externally observable behavioral manifestations. In museum contexts, such exertion is primarily driven by long-distance walking, sustained standing, and the accumulated cognitive load associated with processing high-density exhibition information, which may ultimately surface as visitors’ unintentional compensatory postures. Accordingly, in this work, “museum fatigue” is operationally defined as a specific state exhibited during the visit, resulting from the combined effects of physiological fatigue and cognitive load, and directly manifested through unconscious compensatory postural behaviors.

The present study takes museum fatigue as its main object of study and examines how this phenomenon develops in complex museum environments. The aim is not to provide a full architectural analysis of a single museum, but to construct and test a quantitative framework that combines HPE, questionnaire-based evaluation, and spatial interventions. Jingzhou Museum, a representative garden-style museum composed of several exhibition halls, courtyards, and outdoor transition spaces, is used as the environment in which the framework is implemented and validated. The case is chosen because the combination of long walking distances, frequent indoor–outdoor transitions, and branched routes makes issues of physical effort and cognitive load particularly evident.

**Figure 2:**
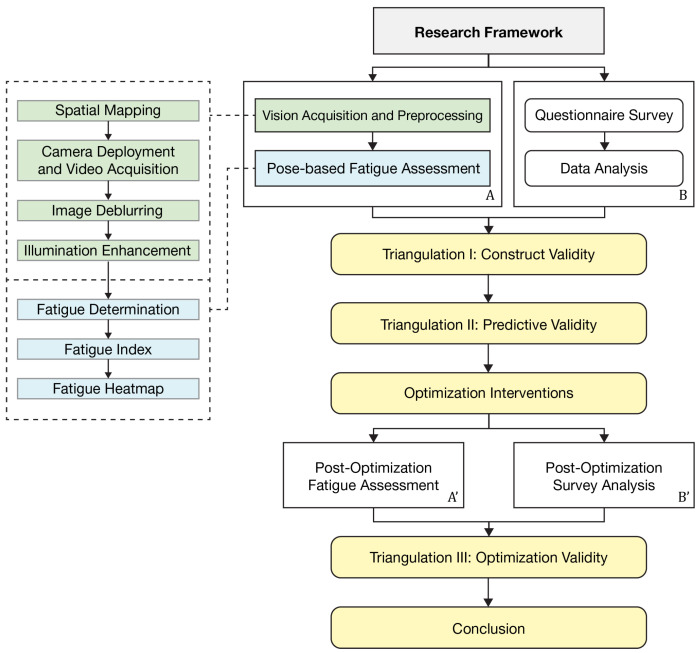
Research framework of the proposed pose-based museum fatigue study. (A) Path A: the computer-vision–based fatigue sensing and spatial mapping pipeline; (B) Path B: the questionnaire-based survey and analysis pipeline. Yellow blocks indicate the three-step triangulation/validity checks (construct, predictive, and optimization validity) that integrate evidence from both paths and inform optimization interventions. A′ and B′ denote the post-optimization repetition of Path A and Path B for evaluation. Arrows show the process sequence; colors distinguish the major modules/stages.

Within this setting, the study builds an overall system architecture for museum fatigue research that links HPE, fatigue assessment, and fatigue mitigation, as shown in Figure 2. The architecture is organized into two parallel paths that provide cross-checks for one another.

Path A starts with Spatial Mapping of the museum plan. Cameras are placed according to an optimal coverage principle, and Camera Deployment and Video Acquisition are carried out to record visitor movement. The recorded videos are decomposed into still image sequences. A Restormer-based model (Version 1.2.0) deep learning algorithm [27] is used for Image Deblurring, and the SG-LLIE model (Version 1.2.0) deep learning algorithm [28] performs Illumination Enhancement, which together improves the quality of the visual data. On the preprocessed images, OpenPose [29] is applied for computer-vision-based HPE and for extracting skeletal keypoints. These pose features support Fatigue Determination and the computation of a Fatigue Index (FI) at each observation point. The resulting values are then projected back onto the architectural floor plan. In this way, a Fatigue Heatmap is obtained at the level of spatial units, revealing where fatigue tends to concentrate within the museum.

Path B consists of a Questionnaire Survey conducted in parallel with visual data collection. Visitors are asked to report their perceived fatigue and to evaluate the spatial environment, which provides a complementary data dimension for perceived experience. The survey results undergo Data Analysis and are then compared with the outcomes of Path A within a triangulation framework to strengthen the credibility of the study’s conclusions. On the basis of these combined findings, targeted Fatigue Mitigation Strategies are developed and implemented as Optimization Interventions, mainly through the adjustment of rest facilities along the visiting route.

To evaluate the effect of these interventions, both paths are repeated in a post-intervention phase. Spatial Mapping and Camera Deployment are kept unchanged and serve as a comparable spatial baseline, ensuring that the pre-intervention and post-intervention data are comparable. Under the new conditions, the full sequence of tasks in Path A, from Video Acquisition through Image Deblurring, Illumination Enhancement, HPE-based Fatigue Determination, and computation of the Fatigue Index (FI), is carried out again, and a new Fatigue Heatmap is generated. In Path B, the Questionnaire Survey is repeated, followed by the Post Optimization Survey Analysis. Finally, the pre-intervention and post-intervention results are brought together in an Effectiveness Analysis, which closes the loop of the system architecture and supports the overall Conclusion of the study.

### 2.2. Video Data Acquisition

**Figure 3:**
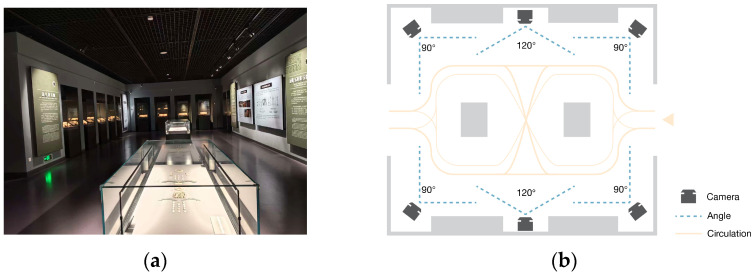
(**a**) Exhibition view; (**b**) camera layout with field-of-view coverage and visitor circulation; grey rectangles indicate exhibition display cases.

Video collection is the first step in the fatigue detection workflow, and its design has a direct impact on the accuracy of subsequent analysis. In large indoor museum spaces with dense visitor flows, video acquisition is often complicated by display case occlusion, changes in crowd density, and uneven illumination, as illustrated in Figure 3a. To address these issues, the present study draws on principles from photogrammetry and video capture to set up a general acquisition scheme for complex spaces. At key nodes within the gallery, multiple cameras are installed to follow a multi-view coverage principle and to reduce occlusion and blind zones. The horizontal field of view of each camera is kept between 90° and 120°, which offers a practical balance between wide-area monitoring and control of geometric distortion, as shown in Figure 3b [30].

Camera coverage rate and blind spot proportion are used as indicators for assessing alternative layouts [31]. The optimization goal is to satisfy basic redundancy requirements while achieving the highest possible coverage with the smallest feasible number of cameras.

After data collection, the continuous video stream is decomposed into a sequence of still images. These images undergo necessary preprocessing so that they meet the input requirements of the subsequent deep-learning-based HPE model.

### 2.3. Image Processing Pipeline

Before conducting the fatigue-related analysis, the image data are first cleaned through a preprocessing step. Frames extracted from the videos often contain motion blur and low or uneven lighting, so deblurring [32] and illumination enhancement [33] are applied in advance to obtain clearer and brighter inputs. These steps provide more stable and reliable image data under complex acquisition conditions and create a better basis for fatigue feature extraction.

In the museum environment, visitor movement, small camera shakes, and nonuniform lighting frequently led to various degrees of motion blur in the frames extracted from the recorded videos. Restormer, proposed by Zamir et al., has shown strong performance on image deblurring tasks [27]. It achieves high-quality restoration with relatively modest parameter count and computational cost, and is particularly suitable for high-resolution images degraded by complex blur.

In this study, the Restormer model is adopted to address image deblurring. Unlike conventional CNN-based approaches [32,34], its central design relies on a Transformer block with two key components: the multi-Dconv head transposed attention (MDTA) module and the gated Dconv feed-forward network (GDFN).

**Figure 4:**
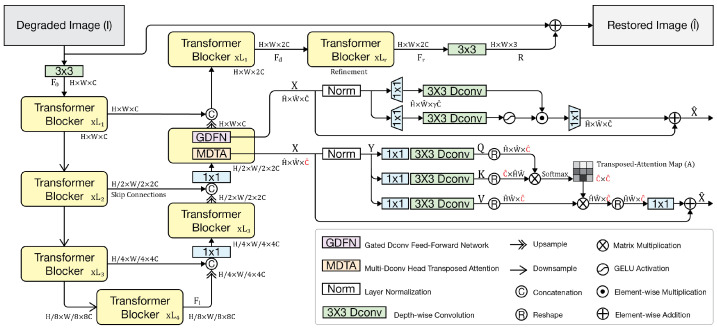
Restormer-based image restoration network architecture. Colored blocks indicate different module categories/stages. Capital letters denote intermediate feature tensors, and red letters annotate tensor dimensions.

The overall Restormer pipeline is illustrated in Figure 4. The MDTA module introduces a cross-channel attention mechanism and shifts the attention operation from the spatial domain to the feature domain, which reduces computational complexity to a linear scale. This design is important for high-resolution museum images, as it allows long-range pixel dependencies to be modeled more efficiently. The GDFN module combines gating mechanisms with depth-wise convolution to strengthen feature representation and suppress redundant channels. By selectively transmitting informative responses and filtering out irrelevant noise through two parallel transformation branches, it performs feature selection and enhancement and further improves texture restoration in the deblurred results.

The explicit formulations of MDTA and GDFN are given as follows:

(1) $$MDTA\left(X\right)=softmax\left(\frac{Q^{⊤}K}{\sqrt{d}}\right)V,$$

where $Q=XW_{Q}$, $K=XW_{K}$, and $V=XW_{V}$; d denotes the feature dimension (per head) used for scaling.

(2) $$GDFN\left(X\right)=\phi \left(XW_{1}\right)⊙\left(XW_{2}\right),$$

where ⊙ denotes element-wise multiplication, and $\phi \left(\cdot \right)$ is a non-linear activation function (e.g., GELU).

The pseudocode is provided in Algorithm 1.
**Algorithm 1.**  IAQ-Gated Robust Inference against Visual AttacksInput: frames x, weight vector w, threshold τ Output: inference results y Line  Pseudocode 1
       // Step 1: Lightweight IAQ feature extraction
2       function Extract_IAQ_Features(x): 3              
$R_{HF}$ ← DCT_HighFreq_Ratio(x) // High-frequency energy ratio 4               $\sigma_{LC}^{2}$ ← Local_Contrast_Variance(x) // Local contrast instability 5               $V_{Lap}$ ← Laplacian_Variance(x) // Sharpness / blur anomaly 6               ${∆}_{RGB}$ ← Color_Channel_Shift(x) // Cross-channel color deviation 7               $C_{temp}$ ← Temporal_Consistency(x) // Inter-frame consistency score 8               return [$R_{HF}$, $\sigma_{LC}^{2}$, $V_{Lap}$, ${∆}_{RGB}$, $C_{temp}$] 9       end function 10 11     // Step 2: IAQ-based risk scoring and gating 12     function IAQ_Gate(x): 13            
feats ← Extract_IAQ_Features(x) 14            
s ← Normalize(feats) ·w // z-score normalization + linear fusion 15            
if s≥τ then // τ: risk-score threshold for high-risk frames 16                   
return “BYPASS” // High-risk frame: bypass sensitive model
17             else
18                   
return “ROBUST” // Normal frame: use enhanced model 19            
end if 20    
end function 21 22    
// Step 3: Main inference pipeline
23     for all frame $x_{t}$ do 24            
gate ← IAQ_Gate($x_{t}$) 25            
if gate == “ROBUST” then
26                    
y ← Run_ROI_Enhanced_Transformer($x_{t}$) 27             else 28                    
y ← Run_Fallback_Baseline($x_{t}$) 29            
end if 30            
append $y_{t}$ to y 31    
end for 32     return y

In the actual exhibition spaces, natural lighting is mixed with artificial sources, and reflections from display glass, together with big differences between foreground and background, often lead to underexposed images and reduced contrast. Image illumination enhancement has therefore become an important part of preprocessing for vision tasks. Classical enhancement techniques [35] can improve visibility to some extent, but they also tend to introduce color distortion and noticeable shifts in hue.

**Figure 5:**
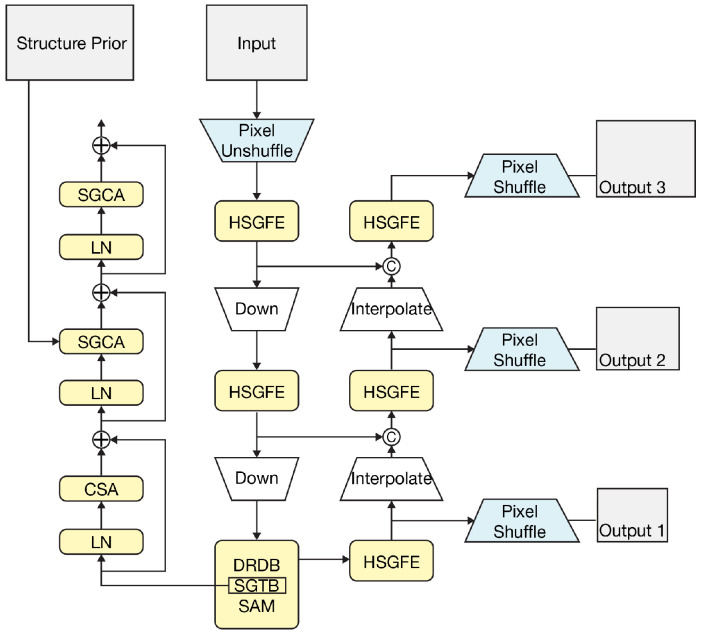
SG-LLIE network architecture for low-light image enhancement. Arrows indicate the dataflow direction. Colored blocks distinguish module categories (e.g., yellow: feature/attention modules; blue: Pixel Shuffle/Unshuffle-based resolution changes; grey: inputs/outputs). ⊕ indicates element-wise addition (skip connection), and C indicates feature concatenation. “Pixel Shuffle/Unshuffle”, “Down”, and “Interpolate” denote the corresponding resolution changes.

To address these issues, the present study adopts the SG-LLIE model in order to recover realistic illumination and structural detail and thereby improve both overall image quality and the performance of downstream modules. Among recent enhancement methods, the structure-guided low-light image enhancement (SG-LLIE) network introduced by Dong et al. [28] is especially relevant. Rather than using a plain convolutional stack, SG-LLIE adopts a U-Net-type encoder–decoder architecture with multi-scale feature fusion modules (SAM) and a hierarchy of structure-guided representations. Its core component is the structure-guided Transformer block (SGTB), which integrates three mechanisms: channel self-attention (CSA), structure-guided cross-attention (SGCA), and a feed-forward network (FFN). The processing pipeline of SG-LLIE is illustrated in Figure 5.

The formulations of SGTB and SGCA are defined as

(3) $$SGTB\left(X,W\right)=FFN\left(SGCA\left(Attn\left(X\right),W\right)\right)+X,$$

where W denotes the structural prior.

(4) $$SGCA\left(X,W\right)=softmax=\left(\frac{(XW_{Q})(WW_{K}{)}^{⊤}}{\lambda }\right)\left(WW_{V}\right),$$

where $W_{Q}$, $W_{K}$, and $W_{V}$ are learnable projection matrices, and λ is a temperature (scaling) factor.

The pseudocode is provided in Algorithm 2.
**Algorithm 2.** Structure-Guided Low-Light Image Enhancement (SG-LLIE)Input: low-light image $I_{LL}$ Output: enhanced image $I_{NL}$ Line  Pseudocode 1        // Step 1: Structure prior extraction 2        function Extract_Structure_Prior($I_{LL}$): 3                E ← Gaussian_Color_Edge($I_{LL}$) 4                W ← Normalized_Spectral_Gradient(E) 5                $W_{out}$ ← CIConv_Normalization(W) 6                return $W_{out}$ 7        end function 8 9        // Step 2: Hybrid Structure-Guided Feature Extraction (HSGFE) 10      function HSGFE($F_{in}$, $W_{prior}$): 11              $F_{1}$ ← DRDB($F_{in}$) // Local detail extraction 12              $F_{2}$ ← SGTB($F_{1}$, $W_{prior}$) // Structure-guided Transformer 13              $F_{3}$ ← SAM($F_{2}$) // Scale-aware fusion 14              return $F_{3}$ 15      end function 16 17      // Step 3: Structure-Guided Transformer Block (SGTB) 18      function SGTB(F, $W_{prior}$): 19              $F_{csa}$ ← CSA(LN(F)) + F // Channel-wise self-attention 20              Q ← Linear_Proj(Reshape($F_{csa}$)) 21              $K_{p}$ ← Linear_Proj(Reshape($W_{prior}$)) 22              $V_{p}$ ← Linear_Proj(Reshape($W_{prior}$)) 23              A ← softmax($\frac{Q\cdot K_{p}}{\lambda }$) // Structure-guided cross attention 24              $F_{sgca}$ ← Reshape(A·Vp) 25              $F_{out}$ ← FFN(LN($F_{sgca}$)) + $F_{sgca}$ // Feed-forward network 26              return $F_{out}$ 27      end function 28 29      // Step 4: Multi-scale encoder-decoder inference 30      Wprior ← Extract_Structure_Prior($I_{LL}$) 31      for l = 1 to L do 32              $F_{enc}\left[l\right]$ ← HSGFE($F_{enc}\left[l-1\right]$, $W_{prior}$) 33      end for 34      for l = L − 1 downto 0 do 35              $F_{dec}\left[l\right]$ ← HSGFE($F_{dec}\left[l+1\right]$ + SKIP($F_{enc}\left[l\right]$), $W_{prior}$) 36      end for 37      $I_{NL}$ ← Reconstruction_Head($F_{dec}\left[0\right]$) 38      return $I_{NL}$


By exploiting this structure-guided Transformer architecture, the model can represent local structural information with high precision and support effective cross-scale feature interaction. In the present context, it raises image brightness and contrast while preserving structural consistency, so that the subsequent museum fatigue detection module receives clearer and more stable visual input.

### 2.4. Fatigue Recognition Model

On the basis of the research positioning and the operational definition of “museum fatigue” established above, this section further proposes behavioral indicators for fatigue identification and justifies their plausibility and relative levels from a biomechanical perspective, thereby providing a theoretical baseline for the subsequent quantification model and weight assignment. Guided by biomechanical principles and a theoretical analysis of fatigue-related compensatory behaviors, three representative compensatory postures are selected as candidate indicators: pronounced lowering of the body’s center of mass (squatting), hand-supported trunk postures (hands-on-hips), and single-leg load transfer (leg-raising). The indicator selection and its construct rationale are mainly supported by three considerations: (1) physiological relevance—these postures correspond biomechanically to active unloading of primary load-bearing muscle groups (lower limbs and the lumbar–back region) and are common responses to muscle fatigue following prolonged standing or walking; (2) temporal covariation with “exertion”—during long-duration, low-intensity visiting activities, their occurrence frequency and duration are expected to be positively associated with exertion-related variables such as visiting time and walking distance; and (3) observability and operability—compared with subtle postural adjustments, these actions have more salient visual characteristics and can be detected more robustly in naturalistic museum settings; combined with rule-based criteria such as duration thresholds, they can further improve the specificity of posture recognition results when used for fatigue determination. Building on this justification framework, we further differentiate the load characteristics of the three postures and establish a theoretical baseline for weight allocation.

During prolonged, low-intensity standing or walking, muscular fatigue is often associated with sustained isometric contraction of the lower-limb and trunk muscle groups and the accumulation of postural control demands; to alleviate discomfort, individuals adopt compensatory strategies that shift the center of mass, redistribute joint moments, or alter muscle recruitment patterns [36]. Specifically, the “pronounced lowering of the center of mass” category (e.g., squatting) substantially increases hip, knee, and ankle flexion and is typically accompanied by a sit-to-stand-like phase; it involves large-range, multi-joint motion and load-bearing control, usually requiring higher lower-limb muscle engagement and joint-moment regulation, and can therefore be regarded as a stronger signal of load release/compensation [37]. The “single-leg load transfer” category (e.g., leg-raising) is often manifested as short-duration shifts in the center of mass and alternating weight-bearing, allowing intermittent relaxation of the non-supporting side; although its movement amplitude is relatively limited, it tends to occur with higher frequency during visits, reflecting an immediate adjustment to localized lower-limb discomfort and a “micro-rest” strategy [38,39]. The “hands-on-hips” category primarily modulates the trunk moment environment and trunk stabilization demands by changing upper-limb configuration and trunk posture, and may provide moment assistance to a certain extent; its load-related effect is more prominent at the level of static maintenance and postural control regulation [40,41].

Considering characteristics such as movement amplitude, triggering threshold, and detectability, these postures can be interpreted as reflecting a decreasing trend in the intensity of compensatory load relief, expressed from stronger to weaker as follows: pronounced lowering of the center of mass > single-leg load transfer > hands-on-hips.

**Figure 6:**
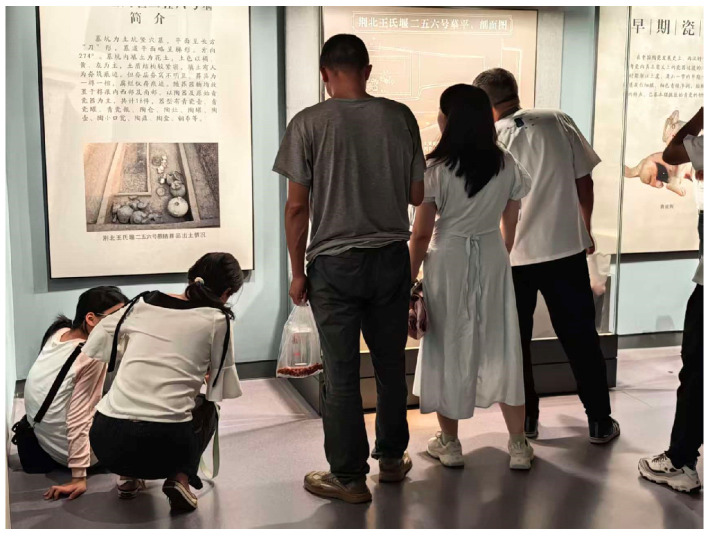
Representative compensatory body postures associated with museum fatigue.

Based on the above operational definition and candidate indicators, dynamic fatigue identification should capture the key process of “accumulation–trigger–recovery” [24,42]. When visitors’ accumulated fatigue during sustained activity exceeds recovery, a transition may be triggered from subtle postural drift to more evident compensatory postures, as shown in Figure 6. The proposed identification model aims to quantify this process using computer vision and aggregate it into computable data at the scale of spatial units.

To follow this gradual change in posture, the study uses a contact-free HPE pipeline built on computer vision. The postures extracted from the videos are mapped onto the museum plan and used to adjust the layout, for example, the position of rest areas, and to design fatigue mitigation measures. The model automatically detects human keypoints and performs analysis of posture, so that changes during the visit can be tracked in a stable way and used as input signals for fatigue analysis [43]. Compared with wearable sensors or questionnaire-based approaches, this setup is non-intrusive, allows continuous data collection, and adapts well to complex spatial configurations, which is important in crowded museums with dynamic flows of visitors.

At the implementation level, OpenPose is used as the core model for fatigue detection. OpenPose is built on a multi-stage convolutional neural network (CNN) and performs bottom-up, real-time detection of human keypoints in single images. The CNN backbone extracts discriminative body-related features from a cluttered background. On this basis, a multi-stage prediction scheme iteratively refines both joint locations and limb association information, and finally outputs the coordinates of the keypoints together with a connected body skeleton [29].

**Figure 7:**
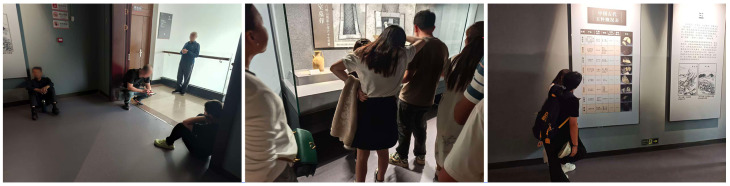
Fatigue-related compensatory postures: squatting, hands-on-hips, and leg-raising.

For fatigue recognition, ten keypoints are selected, covering the shoulders, elbows, hips, knees, and ankles. These joints reflect the loading and support conditions of the main muscle groups. By computing angular changes and displacement amplitudes of the keypoints over time, a mapping is established between posture and fatigue that distinguishes onset, accumulation, and partial recovery. When the deviation of a joint or segment persists beyond a predefined threshold, the visitor is considered to be in a fatigued state. In this phase, compensatory actions such as squatting, placing the hands on the hips, or raising one leg are often observed, as illustrated in Figure 7, as a way to release local muscular pressure and reduce load on the limbs. Detecting and quantifying these typical posture changes allows fatigue-related postures to be identified and measured in a consistent manner.

### 2.5. Fatigue Mitigation Strategies

Although museum fatigue is often noticed first through visible changes in posture, it is, in essence, a cognitively driven process that involves the continuous encoding, interpretation, and integration of exhibition information. High cognitive load not only disperses attention and weakens learning outcomes, but the associated psychological pressure and sustained mental effort also intensify the subjective feeling of fatigue. Therefore, Cognitive Load Theory points to a key principle for mitigation: any effective strategy has to address both the physiological load on the body and the cognitive load on the visitor.

**Figure 8:**
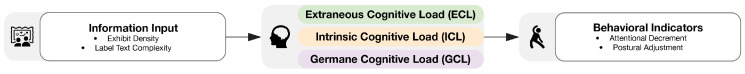
Conceptual links between information input, cognitive load, and behavioral indicators. Color bars distinguish the three cognitive-load components: ECL (extraneous), ICL (intrinsic), and GCL (germane).

Within the methodological framework developed above, the proposed system can identify fatigue states from posture and thus locate areas of high physiological load. Cognitive Load Theory offers a parallel reading of the same spaces. Nodes where dense exhibits, intricate circulation, and repeated decision-making force visitors to process information continuously can be regarded as potential high cognitive load areas. For this reason, posture-based fatigue observed in the experiments is interpreted as an outward behavioral sign of the joint action of physiological and cognitive load. When a visitor adopts compensatory postures such as squatting, standing with hands on the hips, or raising one leg, these body signals not only reflect local muscular discomfort but may also indicate that the cognitive system is working above its comfortable range and is calling for a more integrated form of rest, as illustrated in Figure 8. This combined perspective moves the analysis beyond a purely physical quantification and towards a mechanism that includes mind–body interaction, and it defines a dual optimization target for subsequent mitigation.

In practical terms, high load nodes are adjusted along two dimensions. On the physiological side, additional rest facilities are introduced so that periods of standing and walking are interrupted by short breaks, which ease the static load on the lower limb muscles. On the cognitive side, these pauses provide opportunities for working memory and attentional resources to recover and reset before visitors continue with the exhibition.

### 2.6. Triangulation Framework

Triangulation organizes behavior-based quantification, questionnaire-based experience reports, and pre-/post-optimization re-observation into a progressive chain of evidence, enabling a systematic test of the framework’s explanatory power and effectiveness under real visiting conditions. It comprises three complementary dimensions.

Construct validity examines whether the extracted behavioral proxy signals genuinely reflect perceived fatigue in space. Operationally, the spatial patterns of the fatigue quantification results are compared with questionnaire responses on perceived fatigue intensity, attribution cues, and specific discomfort descriptions, to assess whether meaningful spatial correspondence is observed.

Predictive validity concerns whether the computer-vision pathway provides directional spatial diagnosis and actionable decision support. When questionnaire data indicate visitors’ needs for rest facilities, we test whether the fatigue quantification results can identify the spatial areas where such needs are most concentrated, thereby forming an operational basis for optimization.

Optimization validity is assessed by comparing multi-source changes before and after the intervention. After spatial adjustments, Path A and Path B are repeated; we analyze the pre-/post-intervention correspondence in both trend changes and spatial distributions of the Fatigue Index, and triangulate these findings with questionnaire-based evaluations of intervention effectiveness and perceived experience changes, to examine the practical efficacy of fatigue mitigation and its alignment with experience enhancement.

**Figure 9:**
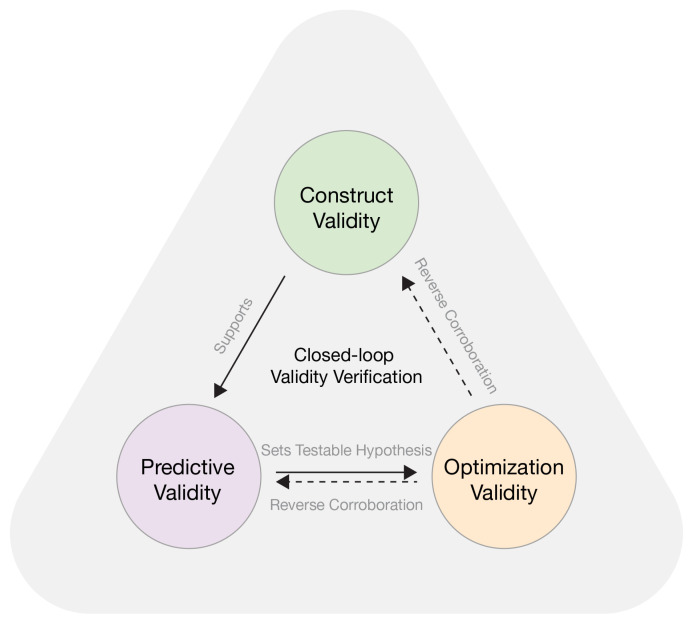
Triangulation framework for closed-loop validity verification across construct validity, predictive validity, and optimization validity.

These three dimensions form a staged relationship: construct validity provides the measurement premise for predictive validity; predictive validity formulates testable spatial optimization hypotheses; and optimization validity verifies these hypotheses through pre-/post-intervention re-observation while offering reverse corroboration of the preceding stages, thereby establishing a closed-loop evidence structure of “perception–diagnosis–optimization–evaluation” as shown in Figure 9.

## 3. Experiments

### 3.1. Overview of the Study Site

**Figure 10:**
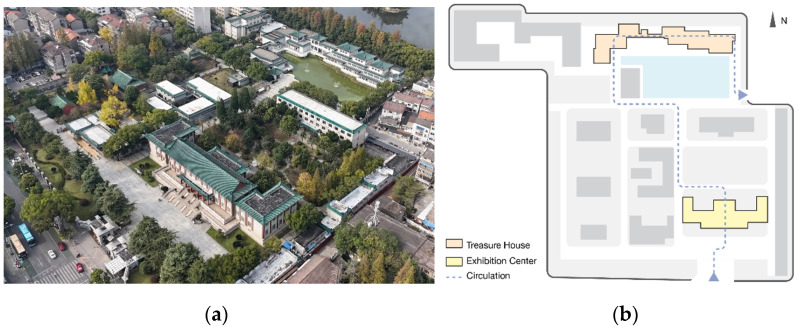
(**a**) Aerial view of the Jingzhou Museum campus. (**b**) Simplified site diagram highlighting the locations and footprints of the Treasure House (orange) and the Exhibition Center (yellow) within the museum grounds; surrounding buildings/landscape elements are shown in gray, the water feature is indicated in light blue, and the primary visitor circulation route is traced by the blue dashed line.

Jingzhou Museum, as shown in Figure 10, stands in the city of Jingzhou in Hubei Province, central China. It is recognized as a national first-class comprehensive museum and takes the material heritage of the ancient Chu state as its main exhibition theme. Its collection includes roughly 196,000 objects [44], featuring Warring States bamboo manuscripts, ritual bronzes, silk fabrics, and lacquered wooden artifacts. Together, they trace the development and artistic achievements of Chu culture and have considerable historical and scholarly value.

Jingzhou Museum is a typical garden-style museum complex. The combination of exhibition halls and landscaped courtyards strengthens the cultural atmosphere and the sense of immersion, but it also produces a complex spatial structure, relatively dense displays, and long walking distances. Under these conditions, visitors are more likely to show bodily signs of fatigue during their visit. In view of these spatial characteristics and the common occurrence of fatigue-related behavior, Jingzhou Museum was chosen as the site for applying the research framework shown in Figure 2.

### 3.2. Pilot Study

To support the quantitative assessment of fatigue-related compensatory postures in museum visits, a pilot study was conducted to obtain prior evidence. Using video clips of individual visitors, we observed spontaneous postural adjustment behaviors and recorded their frequencies of occurrence.

First, to translate compensatory postures into observable and countable indicators, spontaneous postural adjustments in the pre-collected videos were categorized and coded. Frequency statistics showed that, within the “pronounced lowering of the body’s center of mass” category, squatting occurred most frequently; within the “hand-supported trunk” category, hands-on-hips was the most typical; and within the “single-leg load transfer” category, leg-raising was the most common. Together, these three specific postures (squatting, hands-on-hips, and leg-raising) accounted for over 90% of all observed compensatory behaviors and were therefore selected as the core behavioral indicators for subsequent fatigue quantification.

This study adopts a non-intrusive data acquisition approach. With visitor privacy appropriately protected, naturalistic visiting behaviors were captured without interference. The analysis is conducted at the population level, aiming to extract generalizable fatigue-related regularities from repeatable behavioral patterns and their associations with spatial characteristics. This positioning supports the study’s central arguments regarding overall spatial performance and the distribution of fatigue risk across the museum environment.

Given the stable decreasing order of compensatory intensity across the three postures (squatting > leg-raising > hands-on-hips), structural prior parameters were introduced for the integrated fatigue representation. Combining this intensity ordering with the pilot observations, a proportional scheme of 5:3:2 was adopted to formalize the relative contribution gradient, which was then normalized to yield the weight vector (0.5, 0.3, 0.2).

### 3.3. Data Collection Design

In this study, field video recording was confined to the main exhibition spaces of Jingzhou Museum, namely the Exhibition Center and the Treasure House. Other auxiliary buildings were not included. Because the analysis focuses on visitors’ bodily posture and fatigue within exhibition spaces, data were collected only in galleries that support continuous visiting behavior. These two zones carry the principal display program; visitors spend more time there, walk longer distances, and encounter a high exhibit density, which makes them the most likely areas for fatigue-related postures to appear.

**Figure 11:**
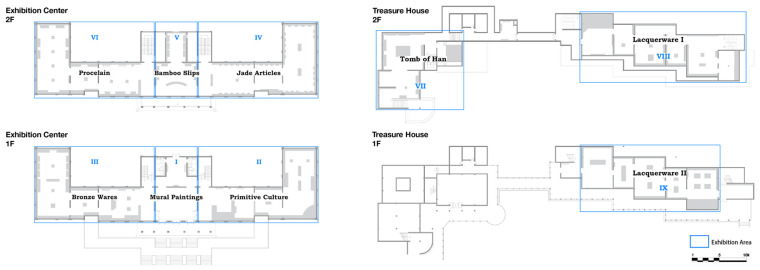
Floor plans of the Exhibition Center and Treasure House (1F and 2F), showing the exhibition areas analyzed in this study. Blue outlines mark the exhibition areas (Galleries I–IX), labeled by collection themes. Thick black lines denote the primary interior spatial enclosure, while gray blocks indicate the exhibition fixtures. Scale bar in meters.

Basic spatial information for the galleries, as shown in Figure 11, was obtained by combining official floor diagrams with on-site surveys and corrections. This combination of official drawings and field measurement is a common procedure in architectural and environmental behavior research [45].

During camera layout, the exhibition spaces are discretized into regular coverage units and a set of candidate camera locations $C=\left\{c_{1},c_{2},\ldots ,c_{M}\right\}$ is defined. These locations are placed at positions where cameras can actually be installed, such as corners, gallery boundaries, and structural supports. Approaches that discretize continuous space and then optimize over candidate sensor positions of this type are widely used in studies on surveillance and sensor deployment [46]. For each candidate location $c_{j}$, the corresponding set of covered units $V\left(c_{j}\right)$ is computed, taking into account the camera field of view, installation height, maximum observation radius, and possible occlusions.

To evaluate a given layout, two indicators are used as core metrics: Covered Area Percentage (CAP) and Uncovered Area Percentage (UAP). Covered Area Percentage is defined in Equation (5) as the proportion of coverage units that are covered by at least r selected cameras:

(5) $$CAP=\frac{\sum_{i=1}^{N}1\left(\sum_{j:g_{i}\in V\left(c_{j}\right)}x_{j}\geq r\right)}{N}\times 100\%.$$

Here, N is the total number of coverage units in the gallery, $g_{i}$ denotes the i-th unit, $V\left(c_{j}\right)$ is the visible region of camera $c_{j}$, $x_{j}\in \left\{0,1\right\}$ indicates whether camera $c_{j}$ is selected, and r is the required level of redundant coverage. Uncovered Area Percentage is given as (6):

(6) UAP=100%−CAP.

**Figure 12:**
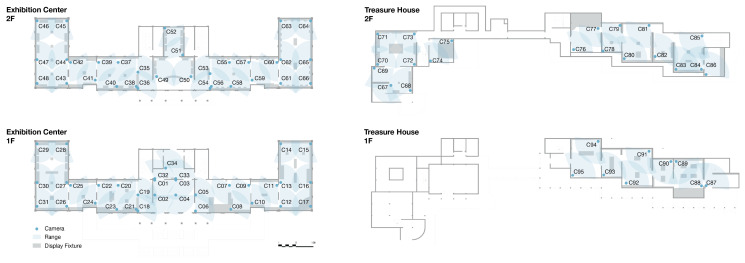
Camera layout and field-of-view coverage.

In modeling the camera deployment, the set of candidate viewpoints is constrained by actual installation conditions, so that each position is feasible with respect to the spatial structure. The selection follows standard guidelines for video surveillance placement and spatial coverage theory [47]. In this study, the usual practice of experience-based placement is made explicit and regularized. Candidate locations are generated only at corners, boundaries, columns, and corridor junctions where installation is realistic, and each candidate is restricted to a feasible range of mounting height, observation radius, and field of view. For every combination of candidate cameras, the proportion of visible area and the proportion of blind area are calculated and compared. On this basis, a layout scheme is chosen that strikes a balance between coverage performance and installation cost, as illustrated in Figure 12.

Based on an accurate three-dimensional model of the galleries, the fields of view of the candidate cameras were simulated under their actual installation parameters. The final deployment achieves a Covered Area Percentage of 93.11%, corresponding to an Uncovered Area Percentage of 6.89%.

During the experiments, cameras continuously recorded high-definition video while visitors moved through the exhibitions in a natural manner. In this study, the resolution was set to 1080p at 30 frames per second. In line with indoor surveillance research that analyzes behavior and crowd flow from continuous video streams [48], this setting preserves both temporal continuity and sufficient motion detail for later analysis. After recording, the videos were split into individual frames, which form the basic input for subsequent image processing and posture feature extraction [49].

In handling the recordings, we followed standard research ethics and data protection rules. Cameras were not used for close-up recording of faces, and the original footage was deleted once skeletal data had been extracted. All data remain within the research team and are not shared externally.

### 3.4. Image Preprocessing Results

**Figure 13:**
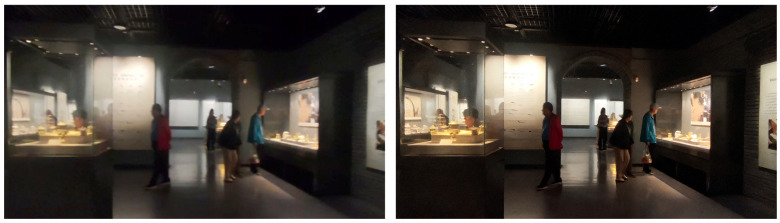
Comparison of the museum scene before (**left**) and after (**right**) image deblurring.

Figure 13 gives a typical pair of frames before and after processing with the Restormer deep learning algorithm. In the unprocessed museum footage, motion during capture blurs the outlines of the visitors, so that the limb segments needed for pose estimation are hard to distinguish. After Restormer processing, the outlines of limbs and trunk become much clearer, and fine details are easier to distinguish.

To assess this improvement in a quantitative way, several standard image quality indices were computed before and after deblurring, as summarized in Table 1. Quantitative indices change in the same direction. Laplacian variance rises from 21.46 to 71.83 (+154.11%), and the mean edge gradient from 9.59 to 24.38 (+234.72%). The Tenengrad score increases from 2933.89 to 4084.87 (+39.23%). RMS contrast shows a smaller gain of 0.97%. Taken together, these changes show that the deblurring step substantially improves the quality of the recorded museum images and provides more reliable input for the subsequent fatigue detection pipeline.

Laplacian Variance [50]:

Let $I\left(x,y\right)$ denote the grayscale intensity image defined on the pixel set Ω, with N pixels (i.e., $N=\left|\Omega \right|$). The Laplacian response is $L\left(x,y\right)=\nabla^{2}I\left(x,y\right)$. The mean Laplacian response is

$$\mu_{L}=\frac{1}{N}\sum_{\left(x,y\right)\in \Omega }L\left(x,y\right).$$

The Laplacian variance is defined as

(7) $$LV=Var\left(L\right)=\frac{1}{N}\sum_{\left(x,y\right)\in \Omega }{\left(L\left(x,y\right)-\mu_{L}\right)}^{2}.$$

Higher LV indicates richer high-frequency content and sharper edges.

Edge Gradient Mean (Sobel) [50]:

Using the Sobel operator, the horizontal and vertical gradients are denoted as $G_{x}(x,y)$ and $G_{y}(x,y)$, respectively. The gradient magnitude is

$$G\left(x,y\right)=\sqrt{{G_{x}(x,y)}^{2}+{G_{y}(x,y)}^{2}}.$$

The mean edge gradient is

(8) $$EGM=\frac{1}{N}\sum_{\left(x,y\right)\in \Omega }G\left(x,y\right).$$

Higher EGM indicates stronger average edge gradients (less blur).

Tenengrad (gradient energy) [51]:

Based on the same Sobel gradients, Tenengrad is defined as the mean squared gradient magnitude:

(9) $$TG=\frac{1}{N}\sum_{\left(x,y\right)\in \Omega }{G_{x}(x,y)}^{2}+{G_{y}(x,y)}^{2}.$$

Higher TG indicates higher gradient energy and clearer edge structures.

RMS Contrast [52]:

Let μ denote the mean intensity of the image:

$$\mu =\frac{1}{N}\sum_{\left(x,y\right)\in \Omega }I\left(x,y\right).$$

The RMS contrast is defined as the root-mean-square deviation from the mean intensity:

(10) $$RMS=\sqrt{\frac{1}{N}\sum_{\left(x,y\right)\in \Omega }{\left(I\left(x,y\right)-\mu \right)}^{2}}.$$

Higher RMS indicates larger global intensity variation (higher contrast).

These findings indicate that the Restormer model deals effectively with motion blur in the museum videos. Visually, the processed images exhibit clearer limb boundaries and more legible postural details, and the quantitative indices also show substantial improvement.

**Figure 14:**
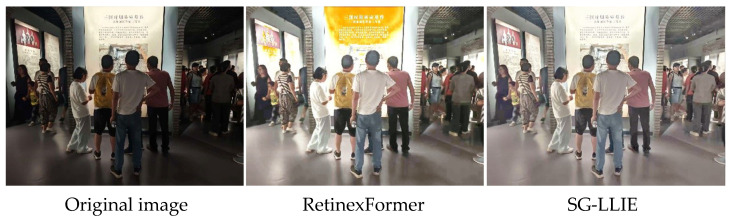
Illumination-enhancement comparison on a museum scene: original, RetinexFormer, and SG-LLIE.

In this experiment, we further evaluated how well the SG-LLIE model enhances illumination in real museum footage. SG-LLIE and RetinexFormer were compared on the low-light enhancement task using both quantitative measures and visual inspection. As shown in Figure 14, the original frames were captured under dim lighting, with evident underexposure, weak structural detail, and poor separation between visitor silhouettes and the surrounding background.

After enhancement with SG-LLIE, overall brightness increases markedly, and the textures around limbs and joints are reconstructed with much greater clarity. In regions where a visitor’s arm overlaps with the torso, details that were previously obscured by low illumination become clearly visible. As shown in Figure 14, RetinexFormer also raises overall brightness, but at the same time introduces a strong warm color cast on the display screen and neighboring highlights. Areas that should be close to white are pushed toward orange, indicating color shift and overenhancement. Such distortions in bright regions hinder faithful color reproduction and may reduce the accuracy of subsequent fatigue motion detection.

To evaluate the illumination enhancement module, a comparative experiment was carried out between SG-LLIE and RetinexFormer [53], a representative low-light enhancement method. Because the showcase screens in this study are expected to appear as nearly colorless neutral white, color shift in the highlight region was quantified by computing the three channel means R mean, G mean, and B mean within the screen ROI, and by introducing $Max\left(R,G,B\right)-Min\left(R,G,B\right)$ as a key index of color distortion. A smaller value indicates a better balance between the three channels and a highlight closer to neutral white, whereas a larger value points to a noticeable color cast. Since the RGB means are computed from 8-bit intensities ($\left[0,255\right]$), the index $D=\max\left(R,G,B\right)-\min\left(R,G,B\right)$ ranges from 0 to 255. Values close to 0 indicate well-balanced channels (nearly neutral white), whereas larger values indicate stronger inter-channel imbalance and a more noticeable color cast.

As summarized in Table 2, within the display screen ROI, the RetinexFormer output gives a value of 149.40 for “Max(R, G, B) − Min(R, G, B)”, which shows that the red channel is strongly amplified relative to the blue channel and produces a marked warm shift; consequently, bright screen areas that were close to white are shifted toward orange. For SG-LLIE, the three-channel means are much closer to each other, and the corresponding “Max(R, G, B) − Min(R, G, B)” is only 14.32. Therefore, the highlight region remains near neutral white, and obvious color distortion is avoided. These results indicate that SG-LLIE maintains more stable colors while increasing brightness, and is a more suitable illumination enhancement component for the museum fatigue detection system.

In addition, to verify the necessity of each component in the proposed image-processing pipeline—namely the Restormer deblurring block and the SG-LLIE illumination-enhancement module—and to quantify their specific contributions to the downstream pose-estimation task, we conducted an ablation study on the same museum-scene test set. As summarized in Table 3, four settings were evaluated: A0 uses raw inputs without any preprocessing; A1 applies Restormer deblurring only; A2 applies SG-LLIE enhancement only; and A3 corresponds to the full pipeline proposed in this work. To measure the impact of preprocessing on OpenPose-based pose recognition, three core metrics were adopted: Mean Conf, Completeness, and Invalid (higher Mean Conf and Completeness indicate better pose quality, while a lower Invalid rate indicates fewer failed/erroneous detections).

The results show that the raw images (A0) are strongly affected by museum-specific motion blur and low-illumination conditions, yielding a relatively low mean confidence (0.6362) and a high invalid rate (0.1360), which indicates substantial risk when performing pose analysis directly on unprocessed video frames. Introducing a single module improves performance to varying degrees. In A1, Restormer reduces the invalid rate from 0.1360 to 0.1204, suggesting that clearer edge and texture cues help suppress erroneous detections under motion blur. In A2, SG-LLIE markedly increases skeleton completeness from 0.7002 to 0.7488, indicating that illumination enhancement is critical for recovering keypoints that would otherwise be missed in shadowed regions.

The full scheme integrating both deblurring and illumination enhancement (A3) achieves the best overall performance. Compared with A0, A3 increases completeness by 12.78% (to 0.7897) and reduces the invalid rate by 14.48% (to 0.1163), confirming the complementary roles of Restormer and SG-LLIE in complex museum environments: the former improves feature fidelity, while the latter improves feature visibility. Such higher-quality preprocessed inputs provide a solid data foundation for the precise fatigue posture determination described in Section 3.4.

All experiments were conducted on an Intel Xeon Platinum 8352 V CPU (16 vCPU) (Santa Clara, CA, USA) with an NVIDIA RTX 4090 GPU (Santa Clara, CA, USA), achieving an average processing speed of 15.1 frames per second (FPS).

### 3.5. Fatigue Determination

At the feature extraction stage, this study relies on OpenPose-based HPE to automatically identify and quantify visitor posture during the museum visit. Using the OpenPose model trained on the COCO dataset [54], a sequence of 17 keypoints is obtained for each person, covering the head, trunk, upper and lower limbs. We note that pose estimation for children can be less reliable due to differences in body proportions and a smaller scale compared to adults [55]. In our implementation, low-confidence or incomplete detections (which may occur more frequently for small-scale subjects) are filtered through the confidence thresholding and required-joint completeness checks described below. Therefore, the current framework is primarily intended for general adult visitor populations, and results involving children should be interpreted with caution. In view of the museum context, three frequently observed compensatory behaviors are taken as the main observable features of fatigue: squatting, standing with hands on the hips, and raising one leg. Kinematic parameters derived from the keypoints are modeled and normalized so that these complex postural patterns can be converted into standardized, comparable indices. The resulting fatigue decision scheme combines several dimensions, as outlined below.

1. Fusion of biomechanical and geometric features. Guided by basic anatomical constraints [56], postural features are described through the spatial relations between skeletal keypoints, especially hip, knee, and ankle joints.
2. Spatial normalization. Pixel coordinates are converted into dimensionless quantities [57] to reduce the influence of image resolution and camera viewpoint on the measurements.
3. Threshold selection. Joint angle thresholds, for example, a knee flexion angle around 120° and an elbow angle between 80° and 120°, are chosen with reference to the ISO 7250 anthropometric standard [58] and to Delp’s work on safe ranges of knee flexion.
4. Real-time computation. The algorithm takes the OpenPose skeleton sequence as input, using the model pre-trained on the COCO dataset to obtain two-dimensional keypoint coordinates. A confidence threshold is applied to remove low-quality detections. In addition, a completeness check is used to handle partial occlusions: for each posture, frames are evaluated only when the required joints are available (e.g., hip–knee–ankle for squatting/leg raising; shoulder–elbow–wrist for upper-limb postures). This avoids posture misclassification caused by missing keypoints under occlusion.

Normalization parameters are adapted to the current image resolution by scaling keypoint coordinates with the frame width/height (and shoulder width when applicable), which helps the method generalize across different camera views and scenes.

5. State confirmation. A posture state is confirmed only when the threshold condition is satisfied for five consecutive frames, which improves the stability of detection. This multi-frame confirmation also suppresses transient errors caused by short-term occlusions and momentary misdetections.

**Figure 15:**
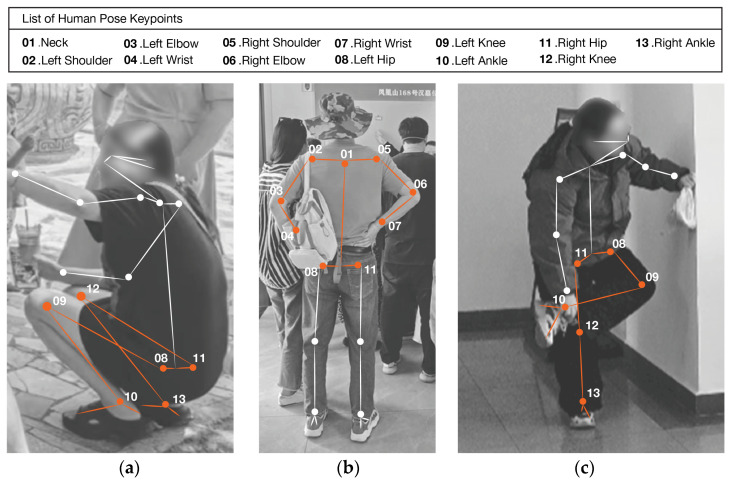
Human pose keypoints extracted from visitor postures using the OpenPose model. (**a**) Example of a squatting posture. (**b**) Example of a hands-on-hips posture. (**c**) Example of a leg-raising posture (single-leg load transfer). The numbered joints correspond to the keypoint list shown above.

After long periods of standing or walking, visitors often squat to relieve fatigue in the lower limbs. To capture this behavior, the OpenPose COCO skeleton model is used to extract the coordinates of the hip (points 8 and 11), knee (points 9 and 12), and ankle (points 10 and 13) joints, as shown in Figure 15a.

When multiple visitors appear in the same frame, OpenPose outputs multiple skeletons. We process each detected person independently and apply the same confidence/completeness filtering and multi-frame confirmation to each track. Figure 14 shows only one representative skeleton for visual clarity, although multiple individuals may be present in the image.

Low-quality detections are first removed using a confidence threshold so that the remaining keypoints are reliable. The angle formed by the hip–knee–ankle vectors is then computed to obtain the knee flexion angle, and the vertical displacement between the midpoint of the hips and the midpoint of the knees is used to decide whether a squat has occurred. When the knee angle falls below 90°, and the hip midpoint shows a clear downward shift, the frame is labeled as a squat posture.

A squat detection method based on joint geometry is proposed by introducing a normalized ratio between hip and knee height. The procedure is as follows.

1. The vertical coordinate of the knee midpoint is obtained as ($Y_{knee}=\frac{\left(Y_{lk}+Y_{rk}\right)}{2}$), and of the hip midpoint as ($Y_{hip}=\frac{\left(Y_{lh}+Y_{rh}\right)}{2}$).
2. A vertical displacement ratio is then defined as
  (11) $$\delta =\frac{Y_{hip}-Y_{knee}}{H},$$
  where H is the frame height.

3. A dynamic threshold is applied: when δ>0.1 and both knee flexion angles $\theta_{lk}$ and $\theta_{rk}$ are below 120°, the target is regarded as being in a squat. This normalized formulation helps reduce the influence of individual body size differences on the detection result.

Hands-on-hips posture is another common sign of fatigue, typically used to ease the load on the lower back muscles. Its recognition depends on the geometric relations between the trunk and upper limbs. Coordinates are taken for the hips (points 8 and 11), neck (point 1), shoulders (points 2 and 5), elbows (points 3 and 6), and wrists (points 4 and 7), as shown in Figure 15b. The shoulder–elbow–wrist angle is calculated to determine whether the arm is flexed. At the same time, the Euclidean distance between each wrist and the corresponding hip keypoint is measured and normalized by the trunk length from the neck (point 1) to the hip midpoint. When the arm is flexed, and this normalized distance falls below a preset threshold, the visitor is classified as exhibiting a hands-on-hips posture.

This study proposes an anthropometry-based criterion for quantifying the hands-on-hips posture. The scheme includes the following decision parameters.

1. Upper-limb spatial location metric. Based on the definitions of anatomical landmark points for the wrist and hip as stipulated in ISO 7250-1:2017 [58], this paper constructs a normalized index for the horizontal distance between the wrist and the hip in a two-dimensional visual coordinate system. The calculation formula is as follows:
  (12) $$\eta =\frac{\left|X_{wrist}-X_{hip}\right|}{W_{shoulder}},$$
  where η<0.3; it conforms to the biomechanical characteristics of the natural downward extension of the upper limbs. Here, $X_{wrist}\;and\;X_{hip}$ represent the horizontal coordinates of the wrist and hip key points in the image coordinate system, respectively. $W_{shoulder}$ represents the horizontal distance between the key points of both shoulders and is used for individual-scale normalization.
2. Elbow kinematic constraint. Elbow flexion angles θ_land θ_rare are computed from three-dimensional vectors along the humerus, radius, and ulna. Both angles are required to fall within a functional range of {80°, 120°}
3. Torso proportion normalization. A normalized torso length τ=THτ=HT is introduced, where T is the distance from the seventh cervical vertebra to the iliac crest, and H is the frame height. The constraint τ∈(0.3,0.7) is imposed to keep the measurement within a plausible anthropometric range.
4. Anatomical consistency check. The vertical coordinates of the left and right hip joints, $Y_{\mathrm{hipL}}$ and $Y_{\mathrm{hipR}}$, must satisfy $Y_{hipL}<\min\left(Y_{wristL},Y_{wristR}\right),Y_{hipR}<min\left(Y_{wristL},Y_{wristR}\right).$ When all four conditions are met simultaneously $\eta \in (0.3,0.45),\theta_{l}\in \{80{}^{\circ},120{}^{\circ}\}$, $\theta_{r}\in \{80{}^{\circ},120{}^{\circ}\}$, τ∈(0.3,0.7), and $Y_{\mathrm{hipL}}<Y_{\mathrm{wristL}},Y_{\mathrm{hipR}}<Y_{\mathrm{wristR}}$, the system classifies the posture as a valid hands-on-hips pose. The corresponding parameters are computed in real time from the OpenPose skeletal output.

Leg raising is another typical compensatory posture used to relieve lower-limb fatigue. By extracting the 2D coordinates of the hip (keypoints 8 and 11), knee (keypoints 9 and 12), and ankle (keypoints 10 and 13) joints as shown in Figure 15c, we compute the knee joint angles of the supporting leg and the raised leg. To reduce false positives caused by overlapping or crossing legs, we introduce horizontal ankle separation γ as an additional constraint. A leg-raising posture is identified when the ankle of one leg is raised above the contralateral knee, or when a pronounced change in knee angle is observed. This posture typically occurs when visitors stand still or after prolonged viewing, reflecting the onset of localized lower-limb fatigue.

In this study, leg raising is defined as a posture state that satisfies the following three biomechanical criteria, as shown in Figure 15:

1. Unilateral knee flexion angle θ∈(70°,130°)
2. Normalized horizontal distance between the midpoints of the plantar support areas of both feet:
  (13) $$\gamma =\frac{\left|∆x\right|}{W}>0.05$$
  where Δx is the horizontal difference between the left and right ankle joints, and W is the video frame width. The threshold of 0.05 is set to ensure a clear physical separation between the two feet in the horizontal direction.
3. Contralateral knee angle $\theta^{\prime }\leq 15{}^{\circ}$

Accordingly, when a target subject simultaneously meets θ∈(70°,130°), γ>0.05, and $\theta^{\prime }\leq 15{}^{\circ}$, the current posture is classified as leg-raising. A spatial normalization procedure is further applied to reduce the influence of inter-subject body-size differences.

On top of these posture detections, a quantitative Fatigue Index (FI) is constructed by fusing multiple posture features. The procedure is as follows.

1. Weights for compensatory postures. Based on pilot data and direct observation, weight coefficients of 0.5, 0.2, and 0.3 are assigned to squatting, hands-on-hips, and leg-raising postures, respectively, to reflect their different contributions to cumulative fatigue.
2. Computation of the fatigue score. For each individual, the raw fatigue score F is obtained as
  (14) $$F=\sum_{i=1}^{3}w_{i}\times f_{i}$$
  where $w_{i}$ is the weight of posture type i and $f_{i}$ is the duration for which that posture is maintained. A larger value of F indicates stronger cumulative fatigue.
3. Normalization of fatigue values. Pilot experiments give a maximum observed fatigue score of $F_{\max}=20$ and a minimum of $F_{\min}=0$. A min–max normalization is applied,
  (15) $$F_{norm}=\frac{F-F_{min}}{F_{max}-F_{min}}$$
  to map the scores to the interval {0,1}.
4. Calibration of the fatigue threshold. Based on the pilot data, the decision threshold for the normalized fatigue score is set to γ=0.15. When $F_{\mathrm{norm}}\geq \gamma$, the visitor is regarded as being in a fatigued state.

To improve reproducibility, Algorithm 3 summarizes the complete rule-based fatigue determination pipeline from OpenPose keypoint streams. The procedure first filters low-quality detections using confidence thresholding and required-joint completeness checks, then performs per-frame posture recognition for the three compensatory postures (squatting, hands-on-hips, and leg-raising) based on the geometric criteria defined above. To suppress transient errors caused by short-term occlusions or momentary misdetections, a multi-frame state confirmation strategy is applied by requiring m consecutive frames before a posture state is accepted. The confirmed posture durations are accumulated with the predefined weights and normalized to obtain a per-visitor fatigue score $S_{v}$, from which a binary fatigue flag $f_{v}$ is derived using the decision threshold γ.

Pseudocode is provided below:
**Algorithm 3.** Museum Visitor Fatigue Identification: DetectFatigueInput: keypoints stream sequence keypoint_sequence Output: per-visitor fatigue flag $f_{v}$, fatigue score $S_{v}$ Line  Pseudocode 1        $w_{squat}$ ← 0.5; $w_{leg\_lift}$ ← 0.3; $w_{akimbo}$ ← 0.2 2         γ ← 0.15 // Fatigue decision threshold 3         $\tau_{squat}$ ← 0.10; $\theta_{knee}$ ← 120° 4         m ← 5 // Multi-frame confirmation (consecutive frames) 5         H ← Frame_Height; Delta_t ← Duration_Per_Frame 6         $T_{win}$ ← Observation_Window 7         $w_{max}$ ← max($w_{squat}$, $w_{leg\_lift}$, $w_{akimbo}$) 8         $S_{min}$ ← 0; $S_{max}$ ← $w_{max}\cdot T_{win}$ 9 10       function DetectPose($k_{t}$): // Per-frame pose detection (priority: SQUAT > AKIMBO > LEG_LIFT) 11               if IsValid($k_{t}$) == False then // IsValid: Confidence threshold + required-joints completeness 12                      return “NONE” 13               end if 14               hips ← GetJoints($k_{t}$, “Hip”) 15               knees ← GetJoints($k_{t}$, “Knee”) 16               ankles ← GetJoints($k_{t}$, “Ankle”) 17               wrists ← GetJoints($k_{t}$, “Wrist”) 18               elbows ← GetJoints($k_{t}$”Elbow”) 19               shoulders ← GetJoints($k_{t}$, “Shoulder”) 20 21               vertical_ratio ← (hips.y − knees.y) / H 22               knee_angles ← CalculateAngles(hips, knees, ankles) 23               if vertical_ratio > $\tau_{squat}$ ∧ min(knee_angles) < $\theta_{knee}$ then 24                      
return “SQUAT” 25              
end if 26              
if CheckAkimbo(wrists, hips, shoulders, elbows) then 27                      
return “AKIMBO” 28              
end if 29              
if CheckLegLift(knees, ankles) then 30                      
return “LEG_LIFT” 31              
end if 32              
return “NONE” 33       end function 34 35       acc_score ← 0 36       c ← 0 37       $p_{prev}$ ← “NONE” 38       for all $k_{t}$ in keypoint_sequence do 39              
pose ← DetectPose($k_{t}$) 40 41       // Multi-frame confirmation: require m consecutive frames 42              
if pose == “NONE” then 43                      
c ← 0 44                      
pprev ← “NONE” 45              
else if pose == $p_{prev}$ then 46                      
c ← c + 1 47              
else 48                      
c ← 1 49                      
$p_{prev}$ ← pose 50              
end if 51 52              
if c≥m then 53                      
$w_{pose}$ ← WEIGHTS[pose] 54                      
acc_score ← acc_score + $w_{pose}\cdot {∆}_{t}$ 55              
end if 56      end for 57 58      $S_{v}$ ← (acc_score − $S_{min}$) / ($S_{max}$ − $S_{min}$) 59      $S_{v}$ ← min(1, max(0, $S_{v}$)) 60      $f_{v}$ ← ($S_{v}$ ≥ γ) 61      return ($f_{v}$, $S_{v}$)

### 3.6. Fatigue Quantification

Based on the per-visitor outputs $f_{v},\;S_{v}$ obtained from Algorithm 3, fatigue is further aggregated at the spatial-unit level for mapping and diagnosis. Algorithm 4 presents the zone/unit-level aggregation logic: for each spatial unit i, we count the total number of unique visitors $N_{t}$ who appear in that unit and the number of visitors $N_{f}$ who are classified as fatigued while staying in that unit. The Fatigue Index is then computed as $FI_{i}=N_{f}/N_{t}$, which serves as the quantitative input for the subsequent heatmap visualization and pre-/post-intervention comparisons.

Pseudocode is provided below:
**Algorithm 4.** Zone-Level Fatigue IndexInput: video stream V, keypoints stream K (OpenPose), zone assignment stream Z, zone number ${Zone}_{i}$ Output: zone-level fatigue index ${FI}_{i}$ Line  Pseudocode 1        visitors ← CountUniqueVisitors(Z, ${Zone}_{i}$) 2        $N_{t}$ ← |visitors|, $N_{f}$ ← 0 3        if $N_{t}$ == 0 then 4               return 0 5        end if 6        for all v ∈ visitors do 7               ${seq}_{i}$ ← FilterByZone(K[v], Z[v], ${Zone}_{i}$) 8               ($f_{v}$, $S_{v}$) ← DetectFatigue(${seq}_{i}$) 9               if $f_{v}$ == True then 10                   $N_{f}$ ← $N_{f}$ + 1 11               end if 12      end for 13      ${FI}_{i}$ ← $N_{f}$ / $N_{t}$ 14      return ${FI}_{i}$


**Figure 16:**
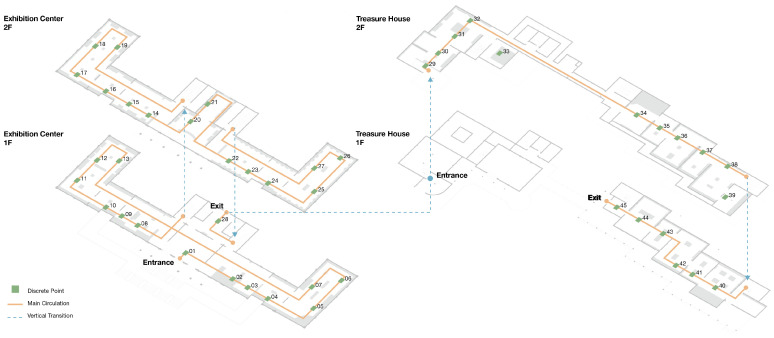
Spatial distribution of discrete observation points along the main circulation. 1F and 2F denote the first and second floors. The numbered labels (01–45) indicate the IDs of the discrete observation points.

The exhibition layout is first partitioned into 45 small analysis cells so that fatigue can be described at the level of local spaces. As shown in Figure 16, each cell is linked to a single discrete point on the plan, which acts as its representative location. Taken together, these points form the reference grid used later to map and display the FI values across the museum. Based on this network of spatial nodes, video is collected for every unit and used as the data source for fatigue quantification.

For the video segment corresponding to each unit, visitors are first detected by the OpenPose model. The total number of visitors who enter that unit is counted and denoted by $N_{t,i}$. The OpenPose-based posture detector is then applied to the same frames to identify fatigue-related postures. If a visitor is judged to be fatigued while staying inside a given unit, that person is labeled as a “fatigued visitor” for that unit. The number of such visitors is recorded as $N_{f,i}$.

The Fatigue Index (FI) for spatial unit i is defined as

(16) $$FI_{i}=\frac{N_{f,i}}{N_{t,i}}$$

which takes values in the interval {0,1}. This index represents the proportion of visitors showing fatigue symptoms among all visitors who have passed through that unit, and thus reflects the relative risk that a given spatial unit induces fatigue, as shown in Table 4.

**Figure 17:**
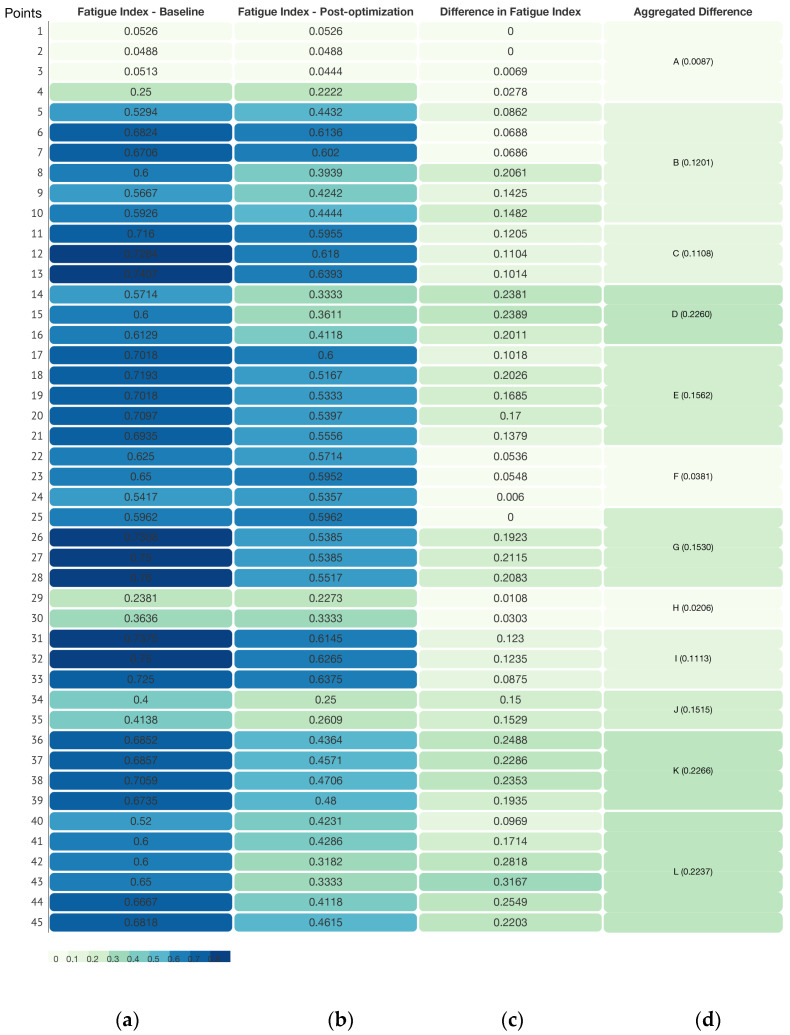
Heatmap of baseline and post-optimization Fatigue Index and reductions at 45 points. (**a**) FI values at baseline. (**b**) FI values after optimization. (**c**) Point-wise FI difference (baseline minus post-optimization). (**d**) Aggregated FI difference summarized by grouped segments of observation points.

**Figure 18:**
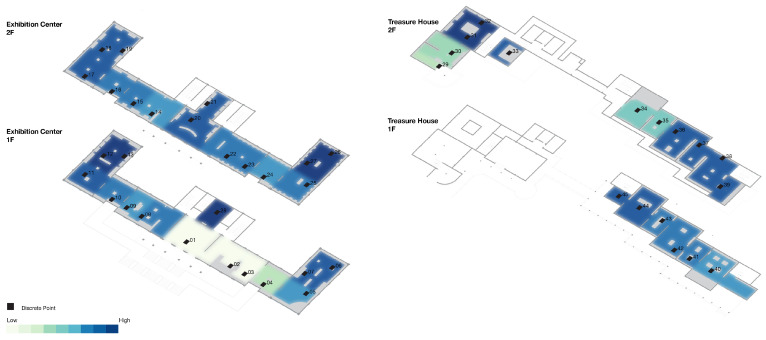
Baseline spatial heatmap of fatigue ratio. The numbered markers (01–45) denote the IDs of discrete observation points used for spatial aggregation; color intensity indicates the fatigue ratio from low to high.

For the 45 spatial units in this study, the computed Fatigue Index (FI) values are classified into three levels on {0, 1} by an equal interval scheme: low fatigue zone, 0 ≤ FI ≤ 0.33; medium fatigue zone, 0.34 ≤ FI ≤ 0.66; and high fatigue zone, 0.67 ≤ FI ≤ 1.00, as shown in Table 5. On this basis, a fatigue index heatmap of the spatial units is produced, as shown in Figure 17a, together with a corresponding map of the discrete representative points, as shown in Figure 18, which together reveal the spatial distribution pattern of fatigue in an intuitive way.

The Fatigue Index (FI) ranges from 0.0488 to 0.7600 (mean = 0.5842), indicating marked fluctuation in fatigue level along the visit route. In Figure 18, the bar colors indicate that FI is concentrated in the middle and upper bands of the scale. Along the first part of the route (points 1–5), FI climbs quickly from 0.0526 to 0.5294. In the compact exhibition zone (points 6–13), it stays at an elevated level and reaches a local peak of 0.7407 at point 13. Along the long circulation corridor (points 14–28), FI fluctuates between 0.5417 and 0.7600, with a local peak around points 19–21. A key buffer appears at point 29, where visitors pass through the garden; fatigue is alleviated, and FI drops to 0.2381 on entering the Treasure House. FI then climbs again from 0.3636 at point 30 to approximately 0.7250–0.7500 at points 31–33. After the designated rest area, FI at points 34–35 falls to 0.4000–0.4138. In the zone with densely arranged, high-value exhibits (points 36–39), FI remains in the range 0.6735–0.7059. The final segment (points 40–45), corresponding to the lacquerware gallery, shows FI values between 0.5200 and 0.6818.

**Figure 19:**
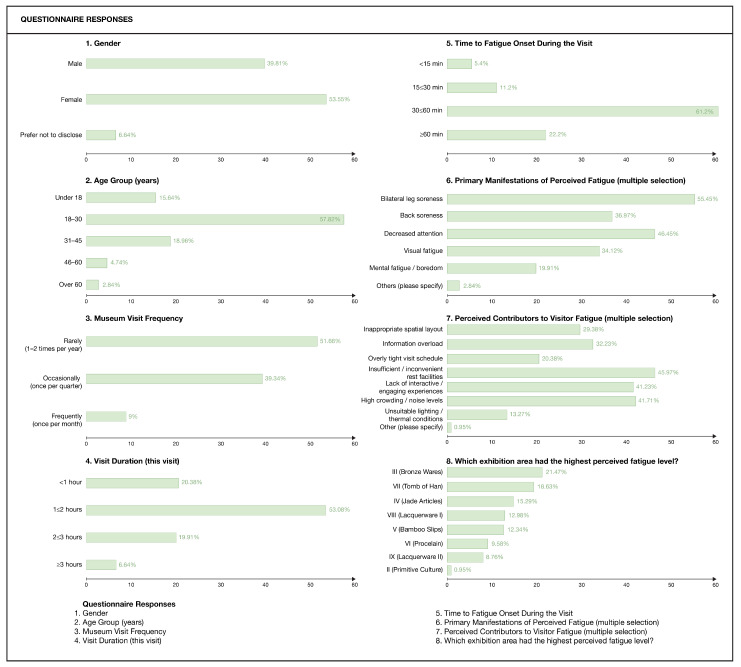
Baseline questionnaire results on visitor fatigue.

To corroborate the fatigue mapping and explore how perceived fatigue relates to spatial factors, a questionnaire survey was conducted, as shown in Figure 19. A total of 233 questionnaires were distributed, and 211 valid responses were collected, giving a valid response rate of 90.56%. Among respondents, 53.55% were female, and 39.81% were male; most were between 18 and 30 years of age (57.82%). Regarding visit frequency, 51.66% reported visiting museums once or twice per year. With respect to visit duration on the survey day, 53.08% stayed for 1–2 h, 20.38% for less than 1 h, 19.91% for 2–3 h, and 6.64% for more than 3 h.

Regarding perceived fatigue, 61.2% of respondents reported that noticeable fatigue first appeared 30–60 min after entering the museum. The main manifestations of perceived fatigue were bilateral leg soreness (55.45%), decreased attention (46.45%), back pain (36.97%), visual fatigue (34.12%), and feelings of boredom or mental fatigue (19.91%). Visitors identified several major contributors to fatigue, including insufficient or poorly located rest facilities (45.97%), high crowd density (41.71%), and excessive informational load of exhibits (32.23%).

As an empirical step for construct validity within the triangulation framework, we examine whether the fatigue-related signals represented by the Fatigue Index (FI) correspond to visitors’ perceived fatigue during the visit. The comparison is conducted across three dimensions: temporal progression, spatial distribution, and symptom–posture correspondence.

1. Temporal progression

The questionnaire data show that 61.2% of respondents first noticed noticeable fatigue 30–60 min after entering the museum. Consistently, the spatial-unit fatigue data indicate that along the initial segment of the visiting route (nodes 1–5), FI increases from 0.0526 to 0.5294.

2. Spatial distribution

In the questionnaire, visitors identified the main fatigue contributors as high crowd density (41.71%) and excessive informational load of exhibits (32.23%). In the spatial-unit fatigue data, FI remains persistently above 0.5667 in the high-information-density galleries (nodes 6–13), while in the long-route exhibition area (nodes 14–28), FI fluctuates at a high level of 0.5417–0.7600.

3. Symptom–posture correspondence

The questionnaire reports the most common physiological symptoms as leg soreness (55.45%) and lower-back discomfort (36.97%). In the posture-recognition statistics, compensatory postures associated with these discomfort regions—squatting and hands-on-hips—also appear with relatively high frequency among fatigue-related postures.

### 3.7. Optimization Experiment

To verify the feasibility and effectiveness of the optimization strategy derived from the predictive validity diagnosis in a real museum setting, we conducted an intervention experiment without altering the original spatial layout or exhibition structure. Instead, local movable facilities were introduced. This strategy directly corresponds to the testable spatial optimization hypothesis formulated under predictive validity: adding rest facilities in high-fatigue-load areas can effectively mitigate visitor fatigue. The experiment deployed temporary resting nodes (i.e., movable benches) at key locations and re-ran the posture detection and fatigue quantification pipeline to collect post-intervention data, thereby empirically evaluating the fatigue-mitigation effect of the proposed intervention.

**Figure 20:**
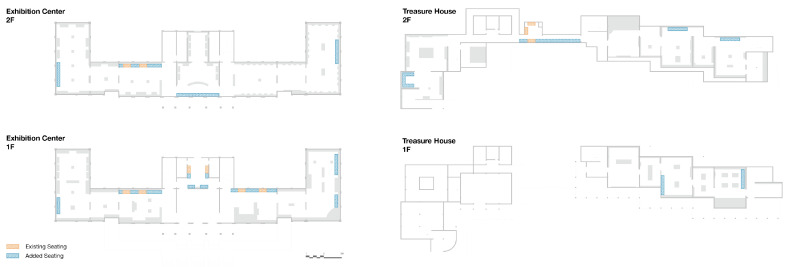
Spatial distribution of existing and added seating.

Intervention locations were determined according to the optimization priority indicated by predictive validity. Based on the spatial fatigue pattern revealed in Figure 18, we selected long-route areas and dense exhibition zones where FI remained persistently high and where rest-related demands from the questionnaire were strongly concentrated, while existing resting/transition zones were retained as behavioral references. In total, 130 movable benches were installed across the intervention areas to form an immediate resting network, as shown in Figure 20. This deployment is intended to directly address the dominant fatigue drivers identified through predictive validity—namely, prolonged standing/walking and elevated cognitive load—and follows the core logic:

(1) Along extended circulation routes, added resting points interrupt continuous walking and are expected to reduce FI values for postures that indicate lower-limb fatigue, such as leg raising and squatting.
(2) In high-density exhibition zones, additional seating allows visitors to sit down, thereby reducing the frequency of postures associated with static loading of the neck and spine, such as hands-on-hips or lateral trunk leaning.
(3) In transitional segments and designated rest areas, extra seating provides opportunities for overall recovery, aiming to lower the combined effect of cognitive and physical fatigue.

**Figure 21:**
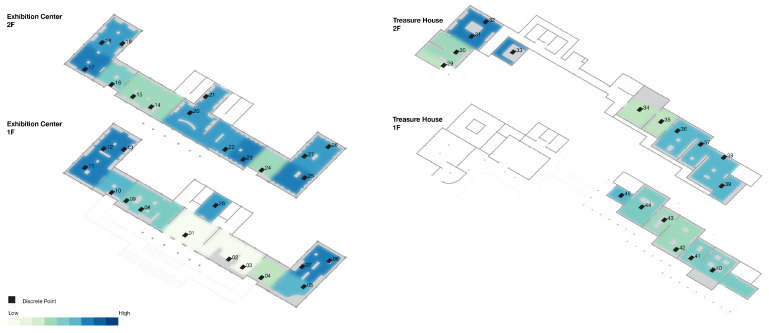
Post-optimization spatial heatmap of fatigue ratio. The numbered markers (01–45) denote the IDs of discrete observation points used for spatial aggregation; color intensity indicates the fatigue ratio from low to high.

After optimization, FI values range from 0.0444 to 0.6393, and the mean FI for the whole museum decreases to 0.4465. Compared with the pre-intervention value of 0.5842, this represents a reduction of 13.78%. The revised heatmaps clearly show that high-fatigue zones contract in size, as shown in Figure 17b and Figure 21.

Key areas all exhibit lower FI values, although the degree of improvement varies across space. In the long circulation segment (points 14–28), FI falls to the interval 0.3333–0.6000. At point 15, for example, FI drops from 0.6000 to 0.3611, a decrease of 23.89%, as shown in Figure 17c. In the dense, high-value exhibition cluster (points 36–39), FI is reduced to 0.4364–0.4800, with point 36 showing a decline of 24.88%. At point 28 near the exit of the main gallery and at point 34 close to a resting zone, the newly added benches lead to reductions of 20.83% and 15.00%, respectively, indicating a clear mitigation effect.

However, some locations show only modest improvement. At points 22 to 25, FI decreases by less than 6%. At the garden transition node (point 29), the difference before and after optimization is negligible, and FI values at points 31 and 32 remain above 0.6145 even after the intervention.

**Figure 22:**
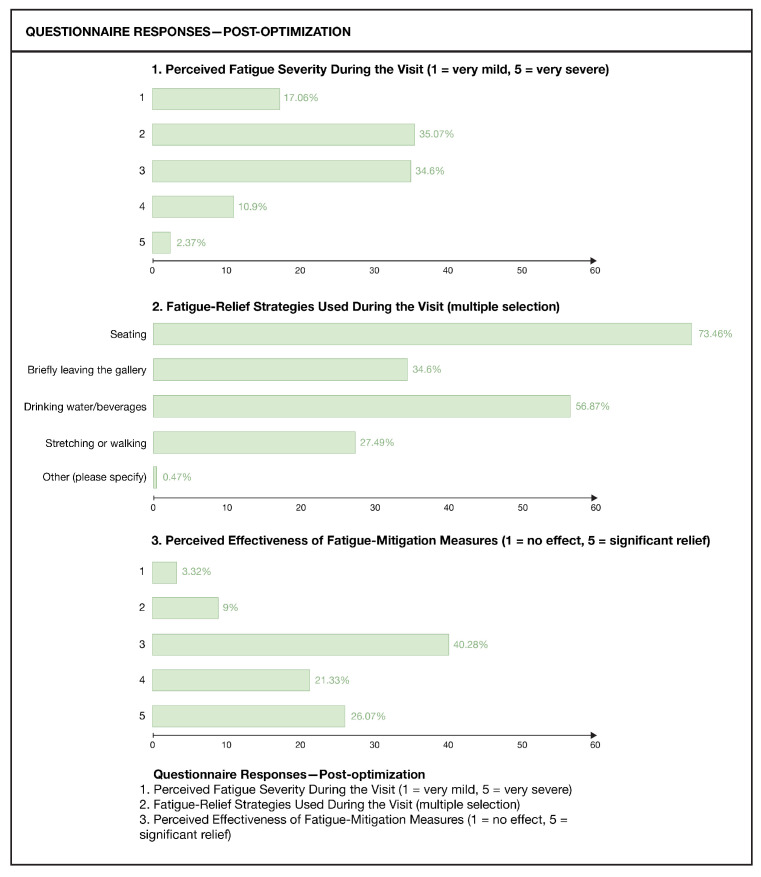
Post-optimization questionnaire results on visitor fatigue.

Visitor self-reports collected after optimization, as summarized in Figure 22, provide an additional perspective on overall fatigue during the visit. For perceived fatigue severity (1 = very mild, 5 = very severe), ratings of two and three account for the largest shares, 35.07% and 34.60%, respectively, giving a combined proportion of 69.67%. A rating of 1 is chosen by 17.06% of respondents, while ratings of four and five are selected by 10.90% and 2.37%.

Regarding fatigue-relief strategies, “seating” is the most frequently used method: 155 respondents (73.46%) report using benches or chairs. Drinking water or other beverages ranks second (120 people, 56.87%), followed by “briefly leaving the gallery” (34.60%) and “stretching or walking around” (27.49%).

Expectations about the effect of additional seating are also positive. In total, 87.68% of respondents believe that more rest facilities can ease fatigue. Among them, 40.28% anticipate a moderate effect (rating 3), 21.33% expect a fairly strong effect (rating 4), and 26.07% anticipate significant relief (rating 5).

As an empirical step for optimization validity within the triangulation framework, post-intervention observations show a decreasing trend in the quantified fatigue results across multiple key spatial units. In the long-route area (nodes 14–28), FI decreases to 0.3333–0.6000; in the high-value and densely exhibited zone (nodes 36–39), FI decreases to 0.4364–0.4800. Notably, node 28 and node 34 exhibit reductions of 20.83% and 15.00%, respectively. Meanwhile, the decrease in areas such as nodes 22–25 remains below 6%, indicating heterogeneous responses across spatial units under the same intervention. In parallel, the post-optimization questionnaire shows that overall self-rated fatigue severity is dominated by ratings of 2 and 3 (69.67% in total) on the five-point scale (1 = very mild, 5 = very severe), and 87.68% of respondents rated the fatigue-mitigation measures as at least moderately effective (effectiveness score ≥ 3). Taken together, the post-intervention reductions in FI and the subjective evaluations align in the direction of improvement, thereby providing optimization-validity evidence for the effectiveness of the current intervention.

## 4. Discussion

### 4.1. Analysis of Fatigue Mitigation

The experimental results indicate that visitor fatigue exhibits a distinctly non-uniform spatial distribution, with high-load spatial units mainly clustered in long circulation segments and high-density exhibition zones. This spatial pattern provides empirical support for the construct validity of the fatigue indicator (FI): elevated FI values tend to occur in areas where visitors are more likely to engage in sustained walking, prolonged standing, and intensive information processing. Based on this spatial diagnosis, the intervention of adding rest facilities demonstrates an overall mitigation tendency in the subsequent evaluation, further reflecting the framework’s closed-loop capability from problem identification to effect feedback. Overall, the experience-based attributions captured in the questionnaire and the behavioral hotspots and dynamic changes revealed by FI form a mutually reinforcing evidence structure, offering strong support for understanding the relationship between spatial design and fatigue perception in museum visits.

**Figure 23:**
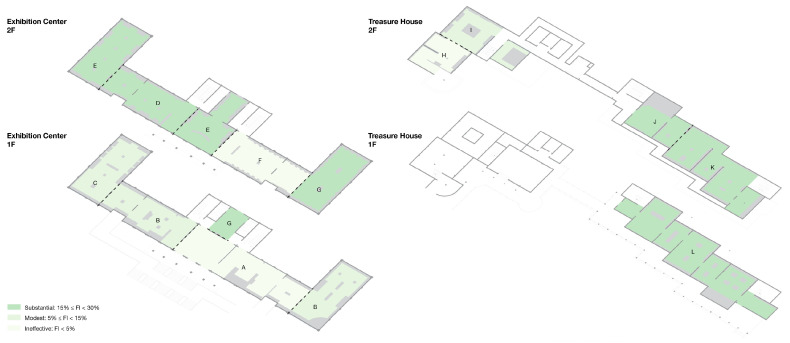
Spatial distribution of fatigue-reduction effectiveness across exhibition zones. The letter labels (A–L) denote the grouped zone segments used for aggregating point-wise FI differences. Colors indicate effectiveness categories based on FI reduction: substantial (15% ≤ FI < 30%), modest (5% ≤ FI < 15%), and ineffective (FI < 5%).

To clarify how changes in fatigue relate to the adequacy of spatial design, the 45 observation points were grouped into 12 trend segments according to fatigue patterns and spatial attributes, as shown in Figure 17d. The corresponding mitigation effects are mapped in Figure 23. Each segment corresponds to a key spatial portion of the visitor route, and differences in mitigation performance directly mirror the quality of the underlying exhibition design:

(A) Entrance baseline segment.

This part of the route covers the initial phase of the visit. No targeted intervention was applied here. Therefore, the data record the initial bodily load and provide a baseline against which the effects in subsequent segments can be interpreted.

(B) Fatigue build-up segment.

Here, the route lengthens, and fatigue begins to accumulate. Some benches were added along the longer corridors, yet visitors are still in a relatively light-load state, and their motivation to use seating is limited [59], so many benches remain underused. The main contribution of the intervention is to slow down the accumulation of fatigue and to lay a buffer for the later high-load stages.

(C) High-intensity exhibition segment.

This area combines dense displays with high information intensity. Visitors must stand and gaze for extended periods, so bodily fatigue and cognitive load rise together [2]. Introducing benches interrupts the ongoing build-up of fatigue: FI values drop in stages, confirming the value of timely rest points in such high-intensity galleries.

(D) Spatial transition segment.

As a key node for vertical circulation, this area naturally passes through a rest zone with additional benches. After optimization, visitors can pause briefly while moving between levels, which helps ease physical load and maintain satisfaction [59].

(E) Complex circulation segment.

**Figure 24:**
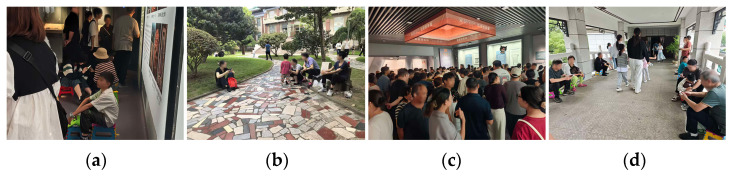
On-site views of fatigue-mitigation measures and visitor resting behavior. (**a**) intricate circulation with dense cases (benches added); (**b**) outdoor garden transition; (**c**) high-value exhibit segment with crowding; (**d**) Treasure House rest segment with increased benches.

In this part of the museum, cases are dense, and the route is intricate; visitors turn and stop frequently, as shown in Figure 24a. Additional benches keep FI values under control, and the mitigation effect is evident. This highlights the importance of local resting facilities when circulation is complex and cognitively demanding.

(F) Facility-deficient segment.

In this segment, the space is relatively enclosed, the display density is high, and the original layout leaves almost no room for seating. FI therefore remains at a mid-to-high level. The issue is not only the absence of benches [60]; continuous case walls and the lack of buffer pockets create a space that constantly draws on visitors’ attention while offering few chances to recover. In such structurally constrained galleries, later optimization has little room to operate; the structural defect of the layout is the root cause of the limited improvement.

(G) End of the long corridor.

This area lies near the end of the main circuit in the exhibition building. Extra benches here give visitors a critical chance to recover. FI values drop markedly, indicating that introducing rest space at the tail of the route helps establish a “recovery–reflection” rhythm before leaving the gallery.

(H) Entrance to the Treasure House.

This segment includes an outdoor garden transition shown in Figure 24b. Even without additional artificial interventions, FI values decline as visitors pass through, revealing the restorative potential of a varied natural environment [61].

(I) High-value exhibit segment.

Here, space is tight, crowds are dense, and high-value artifacts cluster together as shown in Figure 24c. Some extra seating was added, but the mitigation effect is modest. The problem originates in the initial design: the spatial capacity does not match the strong attraction of the exhibits, producing a structural overload. Simple facility supplements cannot compensate for this mismatch; substantial improvements would require spatial reconfiguration and circulation redesign.

(J) Enhanced rest segment.

In Figure 24d, the Treasure House rest area has a larger number of benches. FI values in zone J fall sharply, making it one of the best-performing segments in the entire museum. Beyond confirming the benefit of rest, this result underlines the importance of reserving “optimization interfaces” at the design stage—such as buffer spaces that can later be equipped—to enable low-cost, high-impact adjustments [62].

(K) High-density case segment.

Despite tight spaces and dense cases, the addition of benches still produces a pronounced reduction in fatigue. This successful example shows that in areas where the basic layout is relatively sound, even small “micro-rest nodes” can modulate dwell rhythms and achieve notable mitigation.

(L) Terminal open segment.

This area has lower visitor density and a more generous spatial scale. Combined with the provision of seating, FI values drop substantially. The case suggests that when spatial size is properly controlled, and circulation kept clear from the outset, significant fatigue relief can be obtained with relatively modest investment.

Segment A represents the initial baseline; its low FI is expected and reflects the good starting condition of visitors rather than successful mitigation. Segment H, by contrast, is the desired target condition—a robust “recovery anchor” within the route. The low values in A and H are satisfactory and need little further discussion.

Across the whole system, the mitigation measures reduce the average FI by 13.78%, which, under the grading scheme, falls into the “moderate improvement” band. Different segments respond very differently to the same strategy: in zones D and K, simple facility additions bring large FI reductions of 22.60% and 22.66%, respectively, whereas in zones F and I, FI remains high even after targeted interventions (F: FI = 0.5674 after optimization; I: FI = 0.6262).

This contrast indicates that museum fatigue arises from two levels of causes. The first type of cause is local shortages of support facilities; in such areas, modest interventions—adding benches or rest points—already lead to marked improvements [63], as seen in zones D and K. The second type is embedded in the spatial configuration itself: in zone F, an enclosed plan without buffer space encourages congestion, while in zone I, the limited capacity is mismatched with the strong appeal of the exhibits, producing chronic overload [64]. In such cases, local, non-structural adjustments can only help to a limited extent.

In structurally constrained galleries like F, fatigue remains difficult to alleviate even with added facilities, whereas in better-designed areas like K, simple seating interventions are enough to produce clear benefits. Therefore, the proposed “pose detection–fatigue assessment–fatigue mitigation” framework does more than guide local interventions: by revealing how different spaces respond to the same measure, it also serves as a reverse diagnostic tool for evaluating spatial performance and identifying where deeper design changes are required [65].

### 4.2. Triangulation Analysis

From the perspective of the triangulation framework, the findings can be interpreted across construct validity, predictive validity, and optimization validity.

Construct validity. The questionnaire indicates that the first noticeable fatigue most commonly emerges 30–60 min after entry, with primary manifestations including bilateral leg soreness, decreased attention, and back soreness. Respondents mainly attributed fatigue to insufficient/inconvenient rest conditions, high crowd density, and information overload. These perception-level patterns align with the spatiotemporal form of FI: FI rises rapidly in the early route segment and remains elevated in high-information-density and long-circulation areas, supporting FI as a behavioral construct that externalizes combined physical and cognitive load.

Predictive validity. The location-oriented responses regarding “the exhibition area with the highest perceived fatigue” show clear spatial correspondence with the FI hotspots (e.g., Segment F and Segment I), providing directional evidence for “where intervention is most needed.” In this study, FI operationalizes this demand into a spatial priority ranking and further translates it into a testable hypothesis through the subsequent optimization: after placing movable benches at high-load nodes, the overall FI decreases from 0.5842 to 0.4465 (an absolute decrease of 0.1377), accompanied by a contraction of high-fatigue zones. More importantly, several nodes diagnosed as critical exhibit pronounced reductions exceeding 20% (e.g., nodes 15, 36, and 28), indicating that FI has practical directionality and ranking value for decision support.

Optimization validity. Post-optimization questionnaires show that overall self-rated fatigue severity is dominated by ratings of 2 and 3 (69.67% in total) on the five-point scale (1 = very mild, 5 = very severe). In addition, 73.46% of visitors selected seating as their primary fatigue-relief strategy, and 87.68% rated the mitigation measures as at least moderately effective (effectiveness score ≥ 3). These experience-level evaluations are consistent with the downward trend in FI, suggesting that “the intervention is effective” is not merely a cross-modal agreement but a converging validity outcome.

At the same time, heterogeneous responses provide critical evidence for the framework’s diagnostic depth and closed-loop completeness. In areas such as nodes 22–25, the reduction remains below 6%, and in nodes 31–32, FI remains relatively high after optimization, implying that fatigue drivers in these units may not be dominated by “lack of rest points,” but are more likely related to compounded spatial factors such as crowding, information organization, or route structure. This means the framework can not only identify “high-load nodes” that are improvable via seating-based interventions but can also reverse-identify “differential problem areas” that require alternative prescriptions or structural adjustments. Overall, the study establishes and verifies a complete “perception–diagnosis–optimization–evaluation” loop, and, under the broader context, that an ecological benchmark for museum fatigue remains underdeveloped [25], provides an empirical framework with discriminative diagnostic capacity.

## 5. Conclusions

Using Jingzhou Museum as a case study, this work examines how to detect visitor fatigue and how to design practical mitigation strategies. The main conclusions are as follows.

1. Based on a contact-free computer-vision sensing and analysis framework tailored to museum visitors, this study proposes and validates a pose-based fatigue quantification method, providing initial evidence for its mechanistic feasibility and spatial diagnostic value in real-world settings. Typical compensatory postures can serve as interpretable behavioral proxy signals that translate “fatigue” into spatially computable information without interfering with natural visiting behavior, thereby establishing a quantifiable linkage between visitors’ bodily responses and exhibition-space organization. The framework completes a closed loop in situ—from data acquisition and posture recognition to spatialized outputs—demonstrating its suitability for population-scale assessment and spatial diagnosis in large public environments.
2. Drawing on the combined assessment results and principles from Cognitive Load Theory, a set of fatigue mitigation measures is designed and implemented. The intervention leads to an average reduction of 13.78% in the Fatigue Index (FI) and a corresponding improvement in spatial experience. Importantly, the quantified fatigue results can be directly translated into spatial optimization decisions and subsequently yield testable, measurable improvements after implementation. This advances spatial optimization from experience-based judgment toward an evidence loop of “identify–optimize–re-evaluate”, highlighting the framework’s operational applicability and evaluative capacity.
3. The proposed pose detection–fatigue assessment–fatigue mitigation framework can also be used in reverse as an indicator for diagnosing spatial performance and identifying problem zones in exhibition layouts.

The proposed chain approach provides an indicator system for museum spatial performance evaluation that is grounded in user behavior as evidence. Fatigue-related signals can be interpreted as an externalized representation of spatial friction and use cost, enabling reverse identification of latent issues in layout, circulation, and service configuration, and offering comparable diagnostic references for design iteration and operational management.

Future work can proceed in two directions. First, to enhance generalizability and transferability, the framework should be validated across more museums and diverse visitor populations through cross-context applications. For example, it can be implemented in comparable garden-style museums (e.g., Suzhou Museum) to further test its stability under similar spatial morphologies. In addition, under compliance requirements, more independent reference information (e.g., small-sample physiological measurements) may be introduced to provide more reliable calibration for weight parameters and threshold settings. Second, to improve robustness and reliability, error assessment and algorithmic optimization should be conducted for complex real visiting conditions such as high-density crowds and occlusions, quantifying how factors (e.g., keypoint missingness) affect fatigue determination; and, under privacy-compliant constraints, multi-view and multi-camera collaborative fusion mechanisms can be explored.

## Figures and Tables

**Table 1:** Image quality metrics before and after deblurring (Restormer).

Indicator Name	Numerical Value of Blurred Image	Deblurred Image Values	Improve or Compare
Laplacian Variance ↑	21.46	71.83	154.11%
Edge Gradient Mean ↑	9.59	24.38	234.72%
Tenengrad ↑	2933.89	4084.87	39.23%
RMS Contrast ↑	0.2291	0.2314	0.97%

Definition of image-quality metrics. ↑: The higher this value is, the better.

**Table 2:** RGB channel statistics for RetinexFormer and SG-LLIE.

	R-Mean	G-Mean	B-Mean	Max(R, G, B) − Min(R, G, B)
RetinexFormer	251.04	215.86	101.64	149.40
SG-LLIE	239.32	237.04	225.00	14.32

**Table 3:** Ablation settings and OpenPose performance on the museum test set. ↑ Indicates higher is better; ↓ indicates lower is better. ✓: module enabled; ✗: module disabled.

Setting	Restormer	SG-LLIE	Mean Conf ↑	Completeness ↑	Invalid ↓
A0	✗	✗	0.6362	0.7002	0.1360
A1	✓	✗	0.6384	0.7283	0.1204
A2	✗	✓	0.6390	0.7488	0.1251
A3	✓	✓	0.6434	0.7897	0.1163

**Table 4:** Indicators and spatial statistics for museum fatigue detection.

Indicator Type	Indicator Name	Computation Method	Data Source
Basic statistical indicator	$\mathrm{Total}\;\mathrm{visitors}\;N_{t,i}$	Number of visitors appearing in spatial unit i	Video acquisition plus OpenPose-based detection
Fatigue statistical indicator	$\mathrm{Number}\;\mathrm{of}\;\mathrm{fatigued}\;\mathrm{visitors}\;N_{f,i}$	Count of visitors in unit i whose posture is classified as fatigued (squat, hands on hips, or leg raise)	Posture recognition results
Core quantitative indicator	$\mathrm{Fatigue}\;\mathrm{Index}\;FI_{i}$	$\mathrm{Proportion}\;\mathrm{of}\;\mathrm{fatigued}\;\mathrm{visitors}\;\frac{N_{f,i}}{N_{t,i}}$	Computed from Equation (16)

**Table 5:** Classification of the Fatigue Index $FI_{i}$.

Fatigue Index $FI_{i}$	Fatigue Level	Spatial Characteristics
0.00–0.33	Low fatigue zone	Visitor density moderate; circulation relatively smooth
0.34–0.66	Medium fatigue zone	Local crowding or a relatively dense arrangement of display cases
0.67–1.00	High fatigue zone	Prolonged standing; compensatory body movements occur frequently

## Data Availability

The original data presented in the study are openly available.
